# NOS1AP is a novel molecular target and critical factor in TDP-43 pathology

**DOI:** 10.1093/braincomms/fcac242

**Published:** 2022-09-23

**Authors:** Sara Cappelli, Alida Spalloni, Fabian Feiguin, Giulia Visani, Urša Šušnjar, Anna-Leigh Brown, NYGC ALS Consortium, Hemali Phatnani, Justin Kwan, Dhruv Sareen, James R Broach, Zachary Simmons, Ximena Arcila-Londono, Edward B Lee, Vivianna M Van Deerlin, Neil A Shneider, Ernest Fraenkel, Lyle W Ostrow, Frank Baas, Noah Zaitlen, James D Berry, Andrea Malaspina, Pietro Fratta, Gregory A Cox, Leslie M Thompson, Steve Finkbeiner, Efthimios Dardiotis, Timothy M Miller, Siddharthan Chandran, Suvankar Pal, Eran Hornstein, Daniel J MacGowan, Terry Heiman-Patterson, Molly G Hammell, Nikolaos. A Patsopoulos, Oleg Butovsky, Joshua Dubnau, Avindra Nath, Robert Bowser, Matt Harms, Eleonora Aronica, Mary Poss, Jennifer Phillips-Cremins, John Crary, Nazem Atassi, Dale J Lange, Darius J Adams, Leonidas Stefanis, Marc Gotkine, Robert H Baloh, Suma Babu, Towfique Raj, Sabrina Paganoni, Ophir Shalem, Colin Smith, Bin Zhang, Brent Harris, Iris Broce, Vivian Drory, John Ravits, Corey McMillan, Vilas Menon, Marco De Bardi, Giovanna Borsellino, Maria Secrier, Hemali Phatnani, Maurizio Romano, Pietro Fratta, Patrizia Longone, Emanuele Buratti

**Affiliations:** International Centre for Genetic Engineering and Biotechnology (ICGEB), AREA Science Park, Padriciano 99, 34149 Trieste, Italy; Molecular Neurobiology, Experimental Neuroscience, IRCCS Fondazione Santa Lucia, Via del Fosso di Fiorano 64, 00143 Rome, Italy; Department of Life and Environmental Sciences, University of Cagliari, 09042 Monserrato, Cagliari, Italy; International Centre for Genetic Engineering and Biotechnology (ICGEB), AREA Science Park, Padriciano 99, 34149 Trieste, Italy; International Centre for Genetic Engineering and Biotechnology (ICGEB), AREA Science Park, Padriciano 99, 34149 Trieste, Italy; Department of Neuromuscular Diseases, UCL Queen Square Institute of Neurology, London WC1N 3BG, UK; Neuroimmunology Unit, Experimental Neuroscience, IRCCS Fondazione Santa Lucia, Via Ardeatina 306-354, 00179 Rome, Italy; Neuroimmunology Unit, Experimental Neuroscience, IRCCS Fondazione Santa Lucia, Via Ardeatina 306-354, 00179 Rome, Italy; UCL Genetics Institute, Department of Genetics, Evolution and Environment, University College London, Darwin Building, Gower Street, London WC1E 6BT, UK; Center for Genomics of Neurodegenerative Disease, New York Genome Center, New York, NY 10013, USA; Department of Life Sciences, University of Trieste, Via Licio Giorgieri 5, 34127 Trieste, Italy; Department of Neuromuscular Diseases, UCL Queen Square Institute of Neurology, London WC1N 3BG, UK; UCL Genetics Institute, Department of Genetics, Evolution and Environment, University College London, Darwin Building, Gower Street, London WC1E 6BT, UK; Molecular Neurobiology, Experimental Neuroscience, IRCCS Fondazione Santa Lucia, Via del Fosso di Fiorano 64, 00143 Rome, Italy; International Centre for Genetic Engineering and Biotechnology (ICGEB), AREA Science Park, Padriciano 99, 34149 Trieste, Italy

**Keywords:** ALS, CAPON/NOS1AP, hnRNPs, RNA stability, TDP-43

## Abstract

Many lines of evidence have highlighted the role played by heterogeneous nuclear ribonucleoproteins in amyotrophic lateral sclerosis. In this study, we have aimed to identify transcripts co-regulated by TAR DNA-binding protein 43 kDa and highly conserved heterogeneous nuclear ribonucleoproteins which have been previously shown to regulate TAR DNA-binding protein 43 kDa toxicity (deleted in azoospermia-associated protein 1, heterogeneous nuclear ribonucleoprotein -Q, -D, -K and -U). Using the transcriptome analyses, we have uncovered that *Nitric Oxide Synthase 1 Adaptor Protein* mRNA is a direct TAR DNA-binding protein 43 kDa target, and in flies, its modulation alone can rescue TAR DNA-binding protein 43 kDa pathology. In primary mouse cortical neurons, we show that TAR DNA-binding protein 43 kDa mediated downregulation of *Nitric Oxide Synthase 1 Adaptor Protein* expression strongly affects the NMDA-receptor signalling pathway. In human patients, the downregulation of *Nitric Oxide Synthase 1 Adaptor Protein* mRNA strongly correlates with TAR DNA-binding protein 43 kDa proteinopathy as measured by cryptic *Stathmin-2* and Unc-13 homolog A cryptic exon inclusion. Overall, our results demonstrate that *Nitric Oxide Synthase 1 Adaptor Protein* may represent a novel disease-relevant gene, potentially suitable for the development of new therapeutic strategies.

## Teaser

Cappelli *et al*. reported that Nitric Oxide Synthase 1 Adaptor Protein is a co-regulated transcript of the TAR DNA-binding protein 43 kDa, reduced in amyotrophic lateral sclerosis and frontotemporal lobar degeneration patients with TAR DNA-binding protein 43 kDa pathology. Overall, their results highlight Nitric Oxide Synthase 1 Adaptor Protein as a novel druggable disease-relevant gene in TAR DNA-binding protein 43 kDa-related proteinopathies.

## Graphical Abstract

**Graphical abstract:**
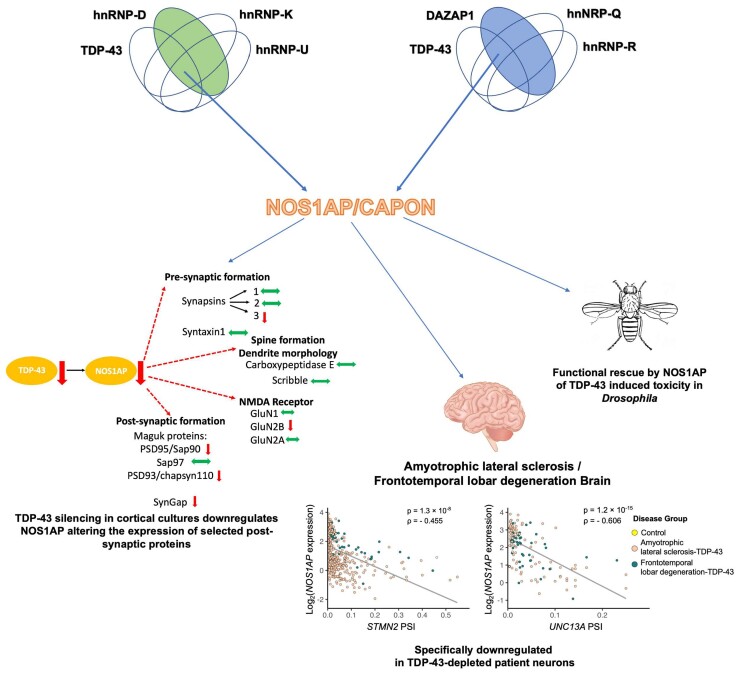

## Introduction

Amyotrophic lateral sclerosis (ALS) is a neurological disorder characterized by the progressive loss of cortical (upper) and spinal cord (lower) motor neurons.^1-3^ The most prevalent form of ALS (90–95% of total cases) is sporadic (sALS), while only 10% of cases are linked to genetic/inherited components attributable to a family history of ALS (fALS).^1,2^ Clinical features of ALS include muscular atrophy and weakness, slurred speech and dysphagia.^4,5^ Neurological symptoms associated with frontotemporal dementia (FTD), such as cognitive, behavioural and language dysfunction, are observed in 5–40% of ALS patients, highlighting an ALS–FTD disease spectrum.^6,7^ From a neuropathological point of view, ALS and FTD are defined by the presence of TAR DNA-binding protein 43 kDa (TDP-43) as the major component of intracellular ubiquitin positive, Tau and α-synuclein negative inclusions.^8^ Pathologic TDP-43 accumulation typically appears in extranuclear compartments, as compared to healthy cells, where TDP-43 is concentrated in the nucleus.^8^ In the heterogeneous FTD syndromes, the presence of TDP-43 inclusions can be categorized into at least five subtypes (A–E), based on the distribution and predominant type of TDP-43-positive structures.^9^ Moreover, its involvement in several neurodegenerative diseases such as Alzheimer’s Disease and Parkinson’s Disease is now recognized and points to TDP-43 as a prominent neuropathological protein.^10-13^

TDP-43 is a 414 amino acid protein implicated in a wide range of cellular processes. As a member of the large family of heterogeneous nuclear ribonucleoproteins (hnRNPs), it is capable of assembling in a complex with pre-messenger RNA (pre-mRNA), controlling all steps of RNA metabolism from synthesis (transcription) to degradation (RNA decay).^14,15^

Over the last decade, other hnRNP proteins have been linked to neurodegenerative disorders and mutations in genes encoding hnRNP-A1, hnRNP-A2/B1, Matrin-3 (MATR3), Fused in Sarcoma (FUS), EWS RNA-binding protein 1 (EWSR1) and TATA-binding protein-associated factor 2N (TAF15) became progressively important in the context of ALS.^16-18^ These disease-associated hnRNP proteins carry common structural features with TDP-43, including a low complexity sequence domain (LCD) required for protein–protein or protein–RNA interaction.^19,20^

The co-ordinated and correct assembly of RNP-complexes mediated by these domains is essential in neurons for the regulation of the expression of RNAs in specific sites within the cells. At the structural level, this assembly is dependent on the ability of hnRNPs to make contact with other RNA-binding proteins that in turn can control and modulate their functions.^21,22^ More recently, the ability of these proteins to assemble in liquid–liquid phase separations (LLPS) has been identified as the strategy to promote membrane less organelles essential for RNA regulation, but also irreversible aggregates found in pathology.^23,24^ For this reason, the neuronal architecture is tightly regulated to avoid perturbations in hnRNP homeostasis that could potentially trigger neurological disorders.^25^

Notably, it is well known that hnRNP proteins can play a modulatory role on TDP-43 function and *vice-versa*, and this has been demonstrated using several cell and animal models.^26-28^ Functional experiments using minigene system carrying Apolipoprotein A2 (*APOA2*) exon 3 have demonstrated that TDP-43 is required for the splicing inhibitor activity of hnRNP-A1.^26^ Additionally, the suppression of cytosine guanine guanine repeat-induced neurotoxicity in a *Drosophila* model of Fragile X-associated Tremor/Ataxia Syndrome (FXTAS) was described to be mediated by the association of TDP-43 with the fly hnRNP-A2/B1 homologues, namely Hrb87F and Hrb98DE.^27^ Very recently, the interaction of TDP-43 with hnRNP-L, PTB/nPTB and hnRNP-A1/A2 was found to affect the inclusion of the exon 17b in the neurotrophic receptor Sortilin 1 (*SORT*1) mRNA, a pathologically relevant splicing event known to be regulated by TDP-43.^28^ Interestingly, this regulation goes both ways, because cytoplasmic aggregation of TDP-43 and dysregulation of RNA metabolism were detected in motor neurons of sALS patients in concomitance with the reduction of nuclear hnRNP-A1 levels.^29^ Moreover, phosphorylation of hnRNP-K by CDK2 has also been shown to regulate TDP-43 cytosolic accumulation.^30^

Taken together, all these evidences suggest that, in addition to TDP-43 pathology, the general expression levels of hnRNPs within neurons (which may depend on individual, cell-specific, environmental or age-related differences) could potentially account for differences in disease onset and progression.^31-34^ From a therapeutic point of view, however, modulating the expression of cellular hnRNPs may present many difficulties, considering the multitude of transcripts that they regulate. As a consequence, a better strategy might be to identify key transcripts co-regulated by hnRNP proteins that we have previously found to worsen or rescue TDP-43-mediated alterations in flies and human neuronal cells.^35,36^ In this work, we have therefore compared transcriptome analyses obtained from human neuroblastoma SH-SY5Y cells silenced for *TARDBP* (encoding TDP-43) and hnRNPs which we previously found were capable of rescuing or worsening TDP-43 toxicity in flies. Specifically, we analysed deleted in azoospermia-associated protein 1 (*DAZAP1*) and *HNRNPQ* (encoding hnRNP-Q), among the ‘rescuing’ hnRNPs, and then we evaluated *HNRNPD* (encoding hnRNP-D), *HNRNPK* (encoding hnRNP-K) and *HNRNPU* (encoding hnRNP-U), among the ‘worsening’ hnRNPs. In addition, to provide better insights into this analysis, we also included the results for *HNRNPR* (encoding hnRNP-R) that, although closely related to hnRNP-Q, was unable to rescue or worsen TDP-43 toxicity in our initial studies.^35,36^

Our results have allowed to narrow down from several hundred genes regulated by each of these hnRNPs, to seven differentially co-regulated genes (*C1orf226*, *CHPF2*, *IGF2*, *IRAK2*, *NOS1AP*, *RNF112* and *UBE2E3*). Interestingly, all these genes are involved in brain functions/neurodegenerative pathways and IGF2 has already been identified as a protective factor in oculomotor neurons of ALS patients.^35^ However, a particularly novel finding of our approach has been the identification of Nitric Oxide Synthase 1 Adaptor Protein (*NOS1AP*, alias *CAPON*) as a novel and most promising target capable of rescuing TDP-43 pathology in neuronal alterations.

## Materials and methods

### Gene knockdown in SH-SY5Y cells

SH-SY5Y cells were silenced against different targets. The siRNA sequences used to silence *TARDBP*, *DAZAP1*, *HNRNPQ* and *HNRNPR* are already described in literature.^35,36^ The siRNA sequences used to knockdown *HNRNPD*, *HNRNPK* and *HNRNPU* are as follows: *HNRNPD* 5′-gauugacgccaguaagaac-3′; *HNRNPK* 5′-aauauuaaggcucuccguaca-3′; and *HNRNPU* 5′-gucacuaacuacaagugga-3′. siRNA against fire-fly luciferase (siLUC) was used as a control: 5′-uaaggcuaugaagagauac-3′. Knockdown efficiency of at least 80% has been achieved through one rounds of silencing using Lipofectamine RNAiMAX (Invitrogen), according to the manufacturer’s instructions. Briefly, the silencing of TDP-43, hnRNP-D, hnRNP-K and hnRNP-U was achieved by seeding 80 × 10^4^ cells in 6-well plates and performing a reverse transfection with a mixture of 150 µl Opti-MEM (Life-Technologies), 3 µl of 40 µM gene-specific siRNA (siTDP-43, siD, siK, siU) or control siRNA and 9 µl of Lipofectamine RNAiMAX reagent. The final siRNA concentration in each plate was 80 nM and one round of silencing was performed at day 0. After 48 h (day 2), cells were collected and prepared for western blot and/or gene expression analysis.

### RNA sequencing analysis of *HNRNPD*, *HNRNPK* and *HNRNPU*

Total RNA was extracted from SH-SY5Y cells treated with siRNA against fire-fly luciferase (control), *HNRNPD*, *HNRNPK* and *HNRNPU* using miRNeasy Kit (Qiagen). Library construction and RNA sequencing was performed by Novogene (https://en.novogene.com/) on three independent experiments obtained for each tested sample.

RNA-seq analysis were performed using Illumina HiSeq NovaSeq 600 instrument. The original raw data from Illumina were transformed to sequenced reads by CASAVA base recognition. Low quality reads (more than 50% reads with nucleotides quality value equal or less than 5 or more than 10% reads with uncertain nucleotides) and reads containing adapter were removed from the analysis. Clean reads were mapped to the reference genome (GRCh38/hg38) using STAR software (v2.5). Differential gene expression analysis was carried out using DEseq2 R package (v2_1.6.3).

The overall distribution of differentially expressed genes (DEGs) were evaluated using the following cut-off: upregulated genes Fold Change (FC) >1.3 and *Padj* < 0.05; downregulated genes FC < 0.7 and *Padj* < 0.05. Volcano plots and Venn diagrams were realized using ggplot2 R package (v3.3.5) and Venny 2.1, respectively. ClusterProfiler package (v3.14.3) from R was also used for Gene ontology (GO) analysis of RNA-seq data. Top 15 enriched ‘Biological Process’ items (*Padj* < 0.05) were considered.

### Protein expression analysis

**Figure 1:**
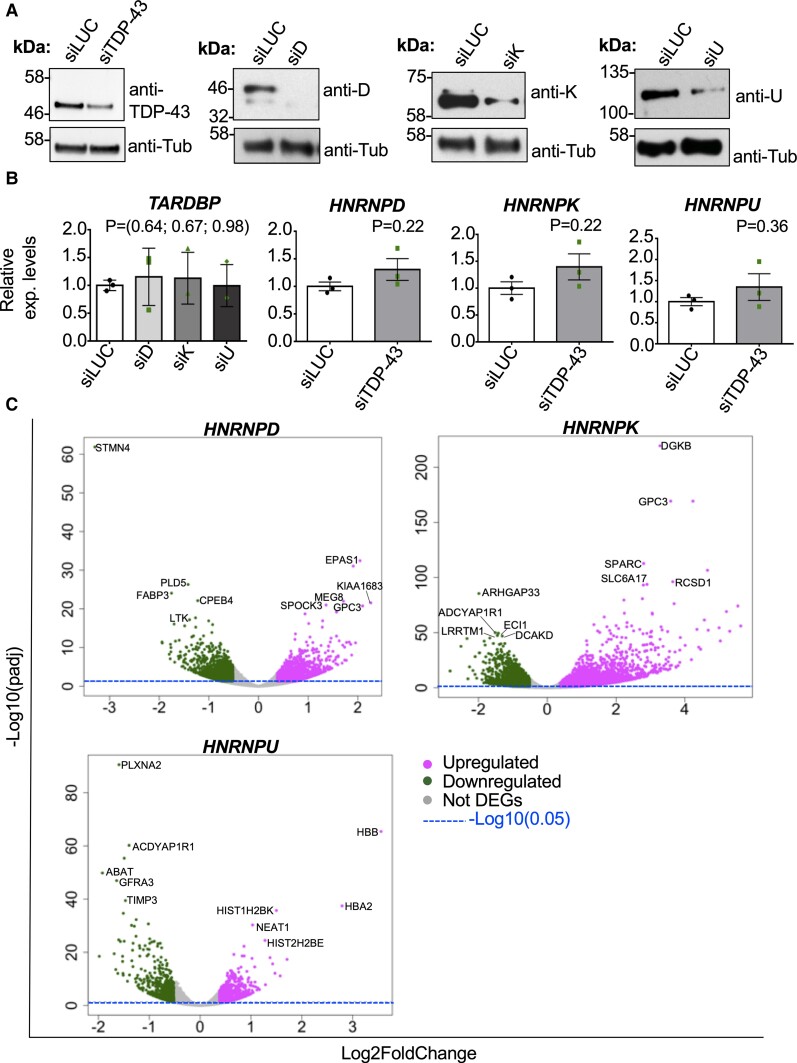
**Effect of hnRNP-D, hnRNP-K and hnRNP-U downregulation in SH-SY5Y cells.** (**A**) Protein expression levels of TDP-43, hnRNP-D, hnRNP-K and hnRNP-U after treatment of SH-SY5Y cells with siRNA against their corresponding genes (siTDP-43, siD, siK, siU) and fire-fly luciferase (siLUC, control). Tubulin was used as loading control. (**B**) RNA expression levels of *TDP-43 (TARDBP)* after silencing of *hnRNP-D (siD)*, *hnRNP-K (siK)* and *hnRNP-U (siU)* and *vice-versa*. Each bar reports the mean ± SEM of 3 independent experiments. Nonparametric un-paired *t-test* was considered for statistical significance (*P < 0.05). (**C**) Whole transcriptome analysis of SH-SY5Y cells depleted for *hnRNP-D*, *hnRNP-K* and *hnRNP-U* respect to the control (luciferase, siLUC). Up- and downregulated genes are reported in the volcano plot as magenta and green dots, respectively. Invariant genes are represented as grey dots. Threshold significance is reported as the horizontal dashed blue line (*Padj* = 0.05). The top 5 up- and downregulated genes are also showed.

Cell pellet was resuspended with a lysis buffer composed of 1 × Phospate Saline Buffer (PBS) supplemented with 1 × Complete Protease Inhibitor Cocktail (Roche) and sonicated at high power with a BioRuptor UCD-200 (Diagenode, Belgium). Protein extract (15–30 µg) was then resuspended in 1 × NuPAGE LDS Sample Buffer (4×) (Thermo Fisher Scientific), boiled at 95°C for 5 min and loaded onto a 10% Bis-Tris 1.5 mm precast gels (Thermo Fisher Scientific). The gel was then electroblotted onto a nitrocellulose membrane (Power Blotter Select Transfer Stacks, Nitrocellulose, Mini Size, Thermo Fisher Scientific) using a Power Blotter–Semi-dry Transfer System (Thermo Fisher Scientific) and incubated with specific primary antibodies (see Supplementary material). The luminescence of the target proteins/loading controls was detected using Luminata Classico Western HRP substrate (Merck Millipore) or SuperSignal West Femto, Trial Kit (Thermo Fisher Scientific). The images were acquired using Alliance 9.7 Western Blot Imaging System (UVITEC, Cambridge), except for the images reported in Fig. 1A regarding hnRNP-D (anti-D), hnRNP-K (anti-K) and hnRNP-U (anti-U), which were developed in darkroom. For each protein expression analysis, an exemplificative western blot image is reported. However, *n* = 3 independent experiments were performed to confirm the data. Uncropped gels are also provided in Supplementary material (see Supplementary Figs 10–13).

### Real-time PCR analysis in SH-SY5Y cells

RNA extraction was performed using miRNeasy Mini Kit (Qiagen) with on column DNA digestion (Qiagen), according to the manufacturer’s instructions. Reverse transcription was carried out at 37°C using random primers (Sigma-Aldrich) and Moloney murine leukemia virus (M-MLV) Reverse Transcriptase (Invitrogen). One microgram was retrotranscribed and the resulting cDNA was diluted 1:10 for quantitative PCR (qPCR) analysis. Housekeeping gene Ribosomal Protein L13a (*RPL13A*) and RNA polymerase II subunit A (*POLR2A*) were used to normalize the results. The target/housekeeping gene sequences are listed in Supplementary material, List of qPCR primers: Supplementary Table 4.

The quantification of gene expression levels reported in Fig. 4A and Fig. 10A and B was performed by quantitative Real-time PCR using PowerUp SYBR Green Master Mix (Applied biosystems) and QuantStudio5 instrument (Applied biosystems). Results were analysed using QuantStudio Design & Analysis software and the mean of relative expression levels ± standard error of the mean (SEM) is reported in the corresponding figures (*n* = 3 or *n* = 4 independent experiments). For each sample, three technical qPCR replicates were considered.

The quantification of *TARDBP*, *HNRNPD*, *HNRNPK* and *HNRNPU* mRNA levels reported in Fig. 1B was performed by using iQ SYBR green supermix (Bio-Rad) on a CFX96 Touch Real-Time PCR Detection System (Bio-Rad). Results were analysed using Bio-Rad CFX Maestro 1.1 software and the mean of relative expression levels ± SEM is reported in the corresponding figures (*n* = 3 independent experiments). For each sample, three technical qPCR replicates were considered. Nonparametric un-paired *t-test* was applied as statistical test (GraphPad Prism software, v6.0). Statistical significance was displayed as *P < 0.05, **P < 0.01 and ***P < 0.0001.

### RNA-Immunoprecipitation (RNA-IP) assay

SH-SY5Y cells were transfected with 16 µg Flag-tagged siRNA resistant TDP-43 plasmid or pRc/CMV control vector using Lipofectamine 3000 transfection reagent (Invitrogen), according to the manufacturer’s instructions. Briefly, 280 × 10^4^ cells were seeded in a 10 mm tissue culture dish and grown under their normal conditions to reach 70–80% confluence on the day of transfection. After 24 h, they were transfected with a mixture of 500 µl Opti-MEM (Life-Technologies), 16 µg of DNA plasmid (control pRc/CMV plasmid or Flag-TDP-43), 30 µl of Lipofectamine 3000 and 32 µl of P3000 reagent (enhancer). Cells were collected and prepared for RNA-IP experiment after 48 h from the transfection. The RNA-IP assay was performed using Imprint RNA Immunoprecipitation (RIP) Kit (Sigma-Aldrich), according to the manufacturer’s instructions. The antibodies used for the reaction are described as follows: 1 µl of anti-mouse IgG antibody produced in rabbit (bridging antibody M7023), 5 µl of mouse monoclonal anti-FLAGM2 (Sigma-Aldrich F1804) (specific target antibody) and 5 µl of IgG from mouse serum (negative control). After 24 h of IP reaction, beads were washed five times and RNA was extracted with EuroGOLD TriFast (Euroclone) as follows in the manufacturer’s instructions. Digestion of genomic DNA was also performed using 5 U/µl DNAse I recombinant, RNase-free (Roche) and the RNA was purified by RNA Clean & Concentrator-5 kit (Zymo Research). Reverse transcription of RNA-IP fractions and 1% Input was carried out at 37°C using random primers (Sigma-Aldrich) and M-MLV Transcriptase (Invitrogen). cDNA was diluted 1:3 for qPCR analysis. The quantification of gene expression levels was performed using PowerUp SYBR Green Master Mix (Applied biosystems) and QuantStudio5 instrument (Applied biosystems). Each RNA-IP fraction Ct value (IgG and anti-Flag) was normalized to the 1% Input RNA fraction Ct value. Mean of relative expression levels ± SEM (*n* = 4 independent experiments) is reported in the corresponding figure. For each sample, three technical qPCR replicates were considered. The expression of Glyceraldehyde-3-phosphate dehydrogenase (*GAPDH*) was also tested as nonspecific target using the primers described in Supplementary material, List of qPCR primers: Supplementary Table 4. Multiple *t-test* was applied as statistical test (GraphPad Prism software, v6.0). Statistical significance was displayed as *P < 0.05.

### mRNA stability assay

SH-SY5Y cells were silenced against TDP-43 or control RNA (siLUC) using Lipofectamine 3000 transfection reagent (Invitrogen). After seeding 18 × 10^4^ cells in a 12-well plate (day 0), cells were silenced twice (at day 1 and 2) in forward transfection using a mixture of 50 µl Opti-MEM (Life-Technologies), 2 µl of 40 µM gene-specific siRNA (or control siRNA) and 3 µl of Lipofectamine 3000. The final siRNA concentration in each plate was 80 nM. After 48 h from the second round of silencing, cells were treated with 5 µg/ml actinomycin D (ActD, Sigma-Aldrich) and collected at 0, 1, 2 and 4 h. Cells were then prepared for western blot and gene expression analysis. Western blot analysis was carried out to check the efficiency of *TARDBP* (TDP-43) knockdown.

The quantification of gene expression levels of *NOS1AP* was performed by qPCR using PowerUp SYBR Green Master Mix (Applied biosystems) and QuantStudio5 instrument (Applied biosystems). Ribosomal Protein L32 (*RPL32*) was considered as reference gene (see Supplementary material, List of qPCR primers: Supplementary Table 4). Data analysis was carried out using QuantStudio Design & Analysis software. The expression of *NOS1AP* was normalized to 0 h for both siLUC and siTDP-43 samples. Mean of relative expression levels ± SEM is reported in the corresponding figure (*n* = 3 independent experiments). For each sample, three technical qPCR replicates were considered. Nonparametric un-paired *t-test* was applied as statistical test (GraphPad Prism software, v6.0). Statistical significance was displayed as *P < 0.05.

### Analysis of *N*-methyl-D-aspartate receptor (NMDAR) signalling pathway in SH-SY5Y cells

To assess the importance of TDP-43-NOS1AP interaction in controlling several transcripts involved in the *N*-methyl-D-aspartate receptor (NMDAR) signalling, SH-SY5Y cells were silenced against a control RNA (siLUC) or TDP-43 (siTDP-43) with or without the overexpression of Flag-tagged NOS1AP plasmid (Sino Biological). Briefly, 32 × 10^5^ cells were seeded in a 6-well plate (day 0). After 24 h (day 1), cells were then silenced using a mixture of 125 µl Opti-MEM (Life-Technologies) and 3 µl of 40 µM gene-specific siRNA (TDP-43 or control siRNA) and 7.5 µl of Lipofectamine 3000 transfection reagent (Invitrogen). The final siRNA concentration in each plate was 80 nM. A second round of silencing was repeated at day 2 with or without the transfection of 4 µg of Flag-tagged NOS1AP expression plasmid (Sino Biological) and 8 µl of P3000 reagent supplied by the Lipofectamine 3000 transfection kit. After 48 h, cells were collected and prepared for gene expression analysis, as described before.

### Patient samples

The NYGC ALS cohort has previously been detailed elsewhere.^37,38^ Sample processing, library preparation, and RNA-seq quality control have already been described in a previous work.^38^

Relative expression levels (Transcripts Per Million) of genes within bulk tissue were adjusted for cell type composition by subtracting the effect of the later (i.e. the proportion of neuron, endothelial cells, oligodendrocytes, astrocytes and microglia) as derived from a multiple regression model. Out of 1349 samples analysed herein, 746 were derived from male and 603 from female individuals. Correlations between transcript were visualized as a correlation matrix plot using the R corrplot package (v.0.84).^39^

### Scoring of *Drosophila* eye phenotypes

Eye degenerations were quantified with an adaptation of the method described by Udai Bhan Pandey.^40^ We examined the phenotypes of minimum 20 fly eyes up to 50 fly eyes. All flies tested were female. During scoring, we hypothetically subdividing each eye in an upper and lower portion for a simpler scoring procedure and checked for the presence of the following features: Omatidial fusion, Single necrosis dots, Middle necrosis patches, Large necrosis patches and Retinal collapse. If the feature was not present, the assigned score was zero, if the feature covered less than 50% of the analysed portion of the eye, the assigned score was 1, while it was 2, if it covered more that 50% of the analysed eye surface. One-way ANOVA with Bonferroni correction and Mann–Whitney were applied as statistical test. In all figures, the values were displayed as the mean ± SEM. Statistical significance was displayed as ***P < 0.001, ****P < 0.0001.

### Cortical cells transfection

Mouse primary cortical cells were prepared as described in the Supplementary material section. At 5 Days *in vitro* (DIV), cortical cells were transfected using Lipofectamine 3000 transfection kit (Invitrogen) with pZac2.1-GFPsh-mTDP-43 plasmid vector; 72 h after transfection (8 DIV) cells were harvested using Accutase (Sigma-Aldrich A6964), collected into conical tubes and fetal bovine serum was added to inhibit Accutase. Cells were then centrifuged for 5 min at 300×g and the pellet was resuspended in 1 × PBS-0.5% bovine serum albumine (BSA). Cell strainers with 50 μm-pore (BD Biosciences) were used to remove debris and cell clumps. Finally, the cells were resuspended into a 1 × PBS-0.5% BSA pre-coated SNAP-cap tube containing 1 mL of PBS-0.5% BSA, DNAase I enzyme (1U/microl Promega Z6011) and 1 µL of propidium iodide solution (PI, Sigma-Aldrich P4864) to identify dead cells; samples were stored on ice up to sorting.

### Cell sorting and isolation of green fluorescent protein positive cortical cells

Mouse primary cortical cells transfected with pZac2.1-GFPsh-mTDP-43 plasmid vector were resuspended in 1 × PBS-0.5% BSA, stained with 1 μl of propidium iodide (PI, SIGMA P4864) for dead cell exclusion and filtered with a 50 μm-pore filter (BD Biosciences) to remove debris and cell clumps. Untransfected cortical cells (green fluorescent protein (GFP)-negative cells) were used to set the GFP positive threshold. GFP positive/PI-negative cells were sorted with a MoFlo Astrios EQ (Beckman Coulter), as shown in Supplementary Fig. 4A–D.

To verify the accuracy of instrument set-up and sorting, GFP positive cells were initially sorted in PBS and the purity check was performed with a Cytoflex flow cytometer (Beckman Coulter). Over 80% purity was consistently obtained (see Supplementary Fig. 4E). Following this control, GFP positive cells were sorted directly into ice-cold lysis buffer (Reliaprep RNA Cell Miniprep System, Promega, Fitchburg, WI, USA), mixed by vortexing and then stored at −80°C until RNA extraction.

Flow cytometry data analysis was performed on FlowJo software v10.8.0 (Becton Dickinson).

### Real-time PCR analysis in cortical cells

Total RNA was extracted from the pZac2.1-GFPsh-mTDP-43 and control cells using the ReliaPrep RNA Cell Miniprep System (Promega). RNA quality (criteria: A260/280 ratio > 1.8) and quantity were assessed using NanoDrop 1000 version 3.7.1 (Thermo Fisher Scientific). cDNA was synthesized at 37°C for 60 min using random primers (0.5 µg), M-MLV Reverse Transcriptase kit (Promega), plus dNTP mix (10 mM, AB) and recombinant RNasin Ribonuclease Inhibitor (25 units, Promega). After optimization of the primers specificity and efficiency (see Supplementary material, List of qPCR primers: Supplementary Table 5), the amount of the mRNA levels of the targeted genes was quantified with the LightCycler 480 System using the SYBR Green I master LightCycler 480 (Roche). Using pool of cDNA from sorting samples, the relative mRNA levels were calculated and then normalized to the housekeeping gene Ribosomal Protein L34 (*RPL34*).^41^ Fold expression was determined using the 2^− ΔCt^. The *RPL34* mRNA levels were similar across pooled cells. Mean Ct values ± SEM was in control cells (*n* = 8 pooled cells): 26 ± 0.6, and in siTDP43 cells (*n* = 8 pooled cells): 26.1 ± 0.46. For statistical analysis after Shapiro–Wilk test (Statistics Kingdom), we used the nonparametric test the two-tailed Mann–Whitney test to compare pZac2.1-GFPsh-mTDP-43 (*n* = 4 pooled cells) and control cells (CTRL) (*n* = 4 pooled cells) for each targeted gene.

### Immunofluorescence microscopy for cortical neurons

At 10 DIV, the cortical cells grown on poly-l-lysine coverslips (three coverslips [13 mm] for each petri dishes [35 mm]) were fixed in 4% PFA/PBS. The coverslips were washed in phosphate-buffer (0.1 M) and permeabilized in 1% Triton X-100 in blocking phosphate-buffer (1% normal donkey serum) for 1 h and probed with the following primary antibodies: rabbit anti-PSD93 (1:200, Thermo Fisher Scientific 34-4700), mouse anti-PSD95 (1:500, Millipore MAB1596), rabbit anti-GluN1 (1:500, Millipore 07-660), rabbit anti-GluN2A (1:200, Millipore 07-632), rabbit anti-GluN2B (1:200, Upstate 06-600) and mouse anti-beta-Tubulin (1:500, Sigma-Aldrich T8660). After washing, the secondary antibodies in normal donkey serum/PBS were incubated at room temperature for 1 h. To visualize the nuclei, the cells were incubated for 30 min with DAPI (4′,6-diamidino-2-phenylindole) in phosphate-buffered solution (1 µg/ml, Sigma-Aldrich).

### Image acquisition and analysis for cortical neurons

The immunofluorescences were acquired in z-stack (zoom = 1). The fluorescence signals were imaged with Axio Observer.Z1/7 inverted fluorescence microscope equipped with an APOX63/1.4 NA oil immersion lens and a filter set for fluorophores in the Cy3, Cy5 (A647), GFP (AF488) and DAPI channels. Images were collected using Zeiss Airyscan LSM800 microscope software. Whole-cells, 8-bit stacks images with 0.3-micron step size were acquired (15–20 planes). Within individual experiments, all images were acquired with identical microscope settings. Brightness and contrast were adjusted equally for all images. For the analysis, the maximum projected images were created. Somata and perisomata ROI areas were defined using beta-Tubulin and GFP fluorescent signal (see Supplementary Fig. 5A and B), and with the Zeiss proprietary software Zen 2.6 Blue edition, we considered the A647 intensity mean values. For quantitative analyses of the intensity levels of each target, the image with the highest immunofluorescence intensity was used. For background subtraction, the ROIs were traced in the area with the minimal A647 intensity mean values. During the analysis, three visual fields were randomly selected in each coverslip, for a total of nine visual fields per dish (representative images in Supplementary Figs 7 and 8). In each field, the immunofluorescence of nontransfected (CTRL) cells, and transfected cells in the scramble (SCR) and siTDP-43 groups was measured as described above. The control groups of each analysed protein were made by the total number of nontransfected cells from the SCR and siTDP-43 groups. For statistical analysis after Shapiro–Wilk test (Statistics Kingdom), the two-tailed Mann–Whitney test was used to compare the pZac2.1-GFPsh-mTDP-43 transfected cells with CTRL cells and with SCR transfected cells (pZac2.1-GFP). The values are obtained from three independent experiments from a minimum of three different fields. The images are presented in the orthogonal maximum intensity projection (MIP).

### Statistical analysis

All statistical analyses were performed with Prism software (GraphPad) version 5.0 or 6.0, as described in each section.

#### Data availability

Data sets discussed in this publication have been deposited in NCBI’s Gene Expression Omnibus^42^ and are accessible through the following GEO Series accession numbers: GSE97262, GSE171090 and GSE193473.

## Results

### RNA-seq analysis of transcripts in neuronal cells

In order to identify the most promising transcripts regulated by several major hnRNP proteins and TDP-43, we have expanded our previous transcriptome analysis obtained from *TARDBP* (TDP-43), *DAZAP1* and *HNRNPQ* (hnRNP-Q) silenced cells^35,36^ to include the analysis of *HNRNPD* (hnRNP-D), *HNRNPK* (hnRNP-K) and *HNRNPU* (hnRNP-U). Using specific RNAi sequences, we have been able to efficiently silence these proteins in SH-SY5Y cells (Fig. 1A) and have demonstrated that the knockdown of *hnRNP-D (siD)*, *hnRNP-K (siK)* and *hnRNP-U (siU)* was not able to affect the *TDP-43 (TARDBP)* mRNA levels and *vice-versa* (Fig. 1B).

**Figure 2:**
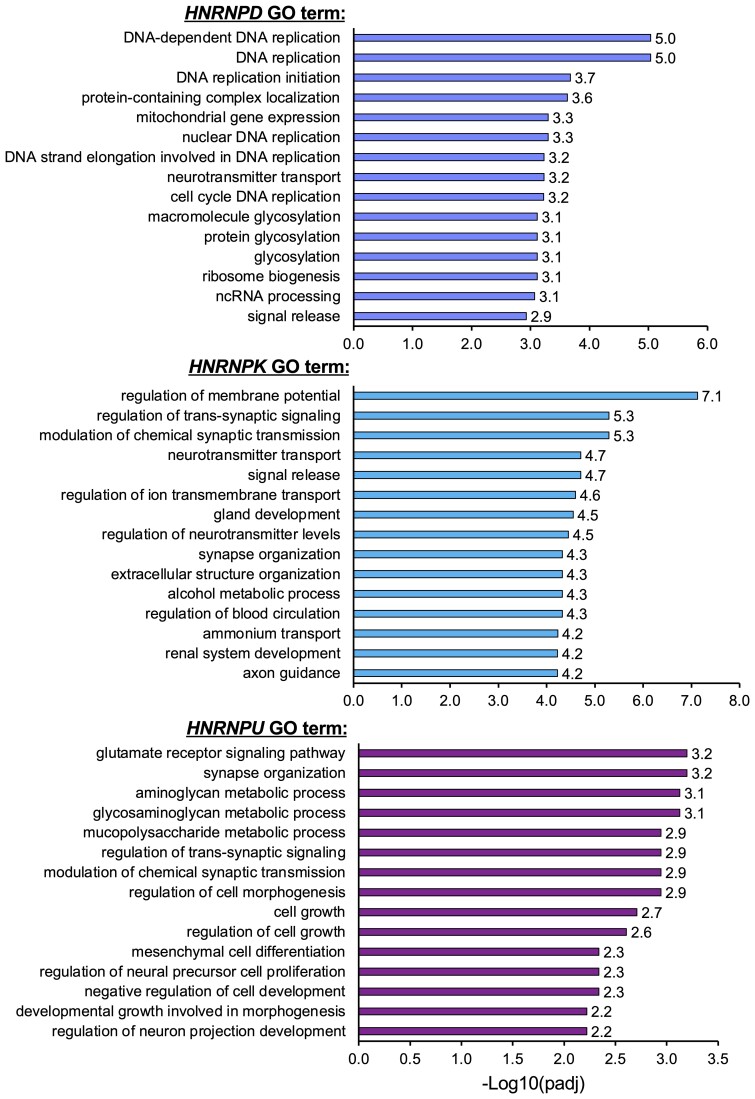
**Gene ontology analysis of RNA-seq data.** Gene ontology analysis were performed using the RNA-seq data obtained from hnRNP-D, hnRNP-K and hnRNP-U depleted cells. The top 15 GO terms from ‘Biological process’ category are presented as horizontal bar with relative −Log10(*P*adj) value.

Regarding the transcriptome analysis, we based our selection of the DEGs on the significance level (*Padj* < 0.05) and the FC cutoffs (upregulation FC > 1.3 and downregulation FC < 0.7) with respect to siLUC treated cells (control). Following these criteria, we found that out of 32 391 analysed genes, 3428 genes were found differentially expressed after *hnRNP-D* silencing (1958 and 1470 genes resulted upregulated and downregulated, respectively). In the case of *hnRNP-K* silenced cells, we detected 6557 DEGs out of 34 575 totally analysed genes (the upregulated and downregulated genes were 4171 and 2386 genes, respectively). Finally, silencing of *hnRNP-U* was able to affect 1667 genes out of the 31 843 analysed genes, of which 860 genes were upregulated and 807 genes were downregulated. A Volcano plot view of these RNA-seq results for each tested hnRNP is reported in Fig. 1C. Upregulated and downregulated genes are highlighted in magenta and green, respectively, and the top 10 DEGs are reported in black. Considering that downregulation of *hnRNP-D*, *hnRNP-K* and *hnRNP-U* in SH-SY5Y cells has never been reported before, we performed a GO analysis for each tested protein, focusing on the top 15 enriched GO classes (*Padj* < 0.05) from the ‘Biological process’ category. Using this approach, we found that these hnRNPs do not seem to involve many overlapping processes (Fig. 2). For example, several genes affected by the silencing of *hnRNP-D* were associated with DNA processing, such as DNA-dependent DNA replication, DNA replication, DNA replication initiation, nuclear DNA replication, DNA strand elongation involved in DNA replication and cell cycle DNA replication. Regarding *hnRNP-K*, we found an enrichment in genes associated with brain functions (e.g. regulation of trans-synaptic signalling, modulation of chemical synaptic transmission, neuron transmitter transport, synapse organization and axon guidance). Finally, *hnRNP-U* silencing was able to modify the expression of genes involved in neuronal signalling such as glutamate receptor signalling pathway, synapse organization, regulation of trans-synaptic signalling, modulation of chemical synaptic transmission, regulation of neuronal precursor cell proliferation and regulation of neuronal projection development. Most importantly, several DEGs such as *CX3CL1*, *SEMA3F*, *OPHN1*, *POLA2*, *MCM10* and *POLD3* were found to be associated with neuronal/brain process and genome stability, that is known to be relevant during neurological disorders.^43^

### Cross-comparison of transcripts co-regulated by hnRNP modifiers of TDP-43 pathology

**Figure 3:**
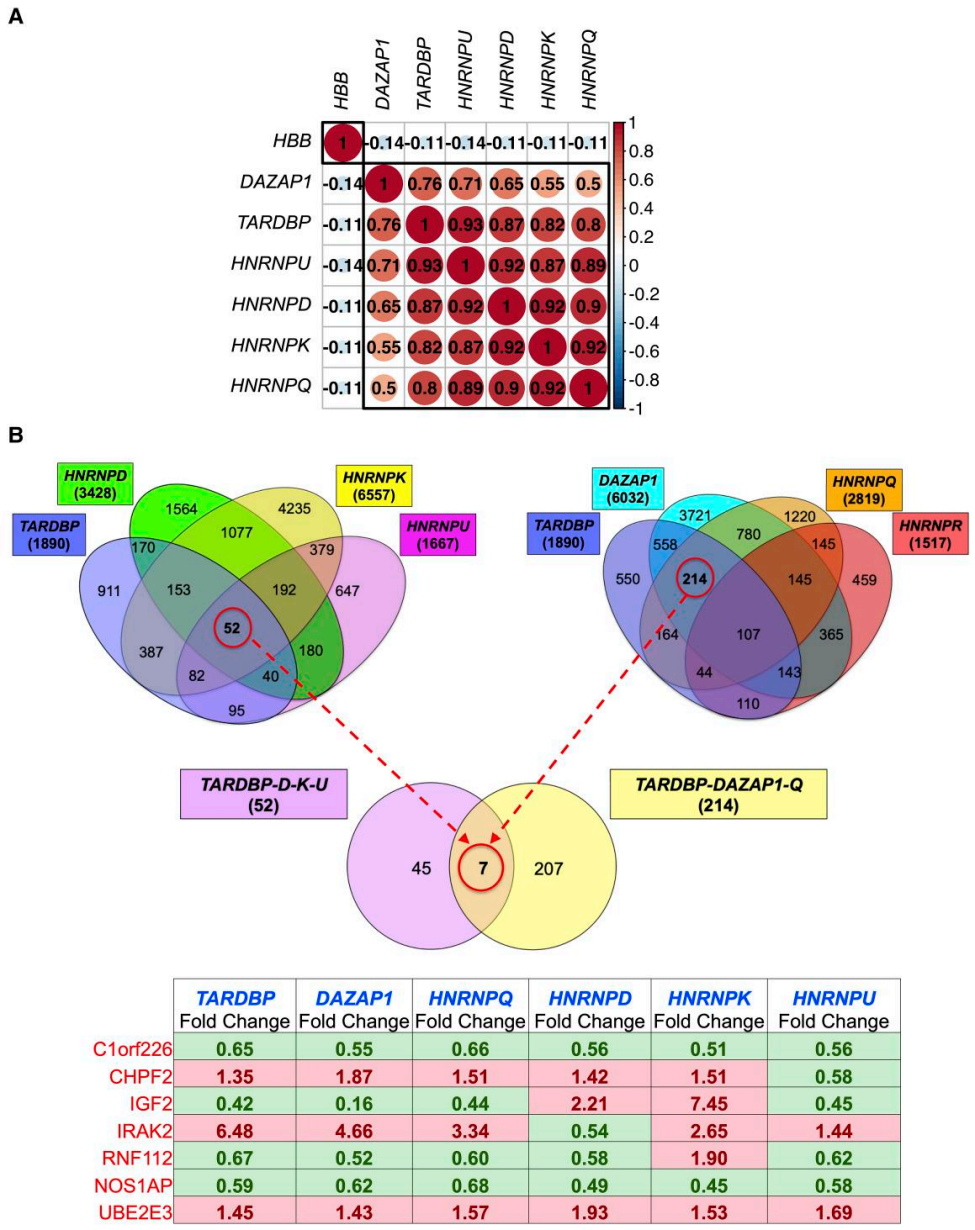
**Cross-comparison of RNA-seq data obtained from TDP-43 and hnRNP depletion.** (**A**) Correlation of *hnRNP* expression in human samples (*n* = 1349) from different brain regions (i.e. cerebellum, cervical spinal cord, lumbar spinal cord, thoracic spinal cord, frontal cortex, hippocampus, motor cortex, occipital cortex and temporal cortex) in ALS/FTLD patients (with or without reported TDP-43 pathology) and healthy controls. Correlation matrix plot visualizes Pearson's correlation coefficients for expression of individual transcripts (i.e. *hnRNPs* and one of their downstream targets: *HBB*) in human brain samples. Gene expression (TPM) was adjusted for cell type composition in balk tissue. Nonsignificant correlations (*P* > 0.05) are crossed out. (**B**) Venn diagram of DEGs obtained from the depletion of TDP-43, *DAZAP1*, *hnRNP-Q*, *hnRNP-R*, *hnRNP-D*, *hnRNP-K* and *hnRNP-U* in SH-SY5Y cells with respect to control (luciferase, siLUC). Data obtained from TDP-43, DAZAP1, hnRNP-Q and hnRNP-R downregulation are currently published^35,36^ and available in GEO for consultation. Red circles are used to highlight DEGs commonly regulated among TDP-43 (TARDBP), hnRNP-D, hnRNP-K and hnRNP-U (52 genes); among TDP-43, DAZAP1 and hnRNP-Q but not nRNP-R (214 genes) and among TDP-43, DAZAP1, hnRNP-Q, hnRNP-D, hnRNP-K and hnRNP-U (7 genes). These latter are also listed in the table with the corresponding Fold Change level and highlighted in red and green colour based on their up- and downregulation, respectively.

Considering the importance of these hnRNPs in affecting TDP-43 function in neurons, we explored their expression levels in the human brain. Interestingly, we found that the expression levels of these factors highly correlate in samples derived from different neuroanatomical regions irrespective of the pathology (i.e. in ALS/FTLD patients and healthy controls) (Fig. 3A). As gene expression levels were adjusted for cell type composition within bulk tissue, we excluded the possibility that high correlations simply reflect the variable proportion of neuronal cells in each sample, along with the fact that hnRNPs are generally abundantly expressed in neurons. However, we observed low association between expression of hnRNPs and a representative downstream target (*HBB*, Fig. 1C volcano plot of hnRNP-U), suggesting that the high correlations shown in Fig. 3A are characteristic of this set of hnRNPs. In addition to the functional data, these findings support our initial intention to combine the results obtained from the knockdown of TDP-43 hnRNP modifiers to obtain deeper insight regarding which transcripts could be relevant for the onset and progression of disorders associated with hnRNPs dysregulation.

As mentioned before, in fact, we have previously identified 214 genes commonly regulated among TDP-43, DAZAP1, hnRNP-Q but not hnRNP-R (Fig. 3B, Supplementary Table 1), which is closely related to hnRNP-Q but does not modify TDP-43 pathology.^35^ Among these genes, we found very promising targets, such as *IGF2*^44^ and *SYT14*.^45^ However, as 214 genes represents still a large number to examine in detail, we further looked at the transcriptome changes induced by the knockdown of *TDP-43* and *hnRNP-D*, *hnRNP-K* and *hnRNP-U*, identifying 52 commonly regulated genes (Fig. 3B). Like the previous comparison, several of these transcripts, such as *CELF5*, *DEPTOR*, *DLG2*, *OPTN* and *STX3* (Supplementary Table 2), have been linked to brain functions and neurological disorders, supporting the fact that these hnRNP proteins can work in a network to regulate at least specific sets of targets. Notably, the cross-comparison of these two data sets, yielded seven commonly regulated transcripts potentially relevant for the development of a therapeutic strategy against TDP-43 pathology, namely *C1orf226* (Chromosome 1 Open Reading Frame 226), *CHPF2* (Chondroitin Polymerizing Factor 2), *IGF2* (Insulin-Like Growth Factor 2), *IRAK2* (Interleukin 1 Receptor-Associated Kinase 2), *RNF112* (Ring Finger Protein 112), *NOS1AP* (Nitric Oxide Synthase 1 Adaptor Protein) and *UBE2E3* (Ubiquitin-Conjugating Enzyme E2 E3) (Fig. 3B, lower Venn diagram).

**Figure 4:**
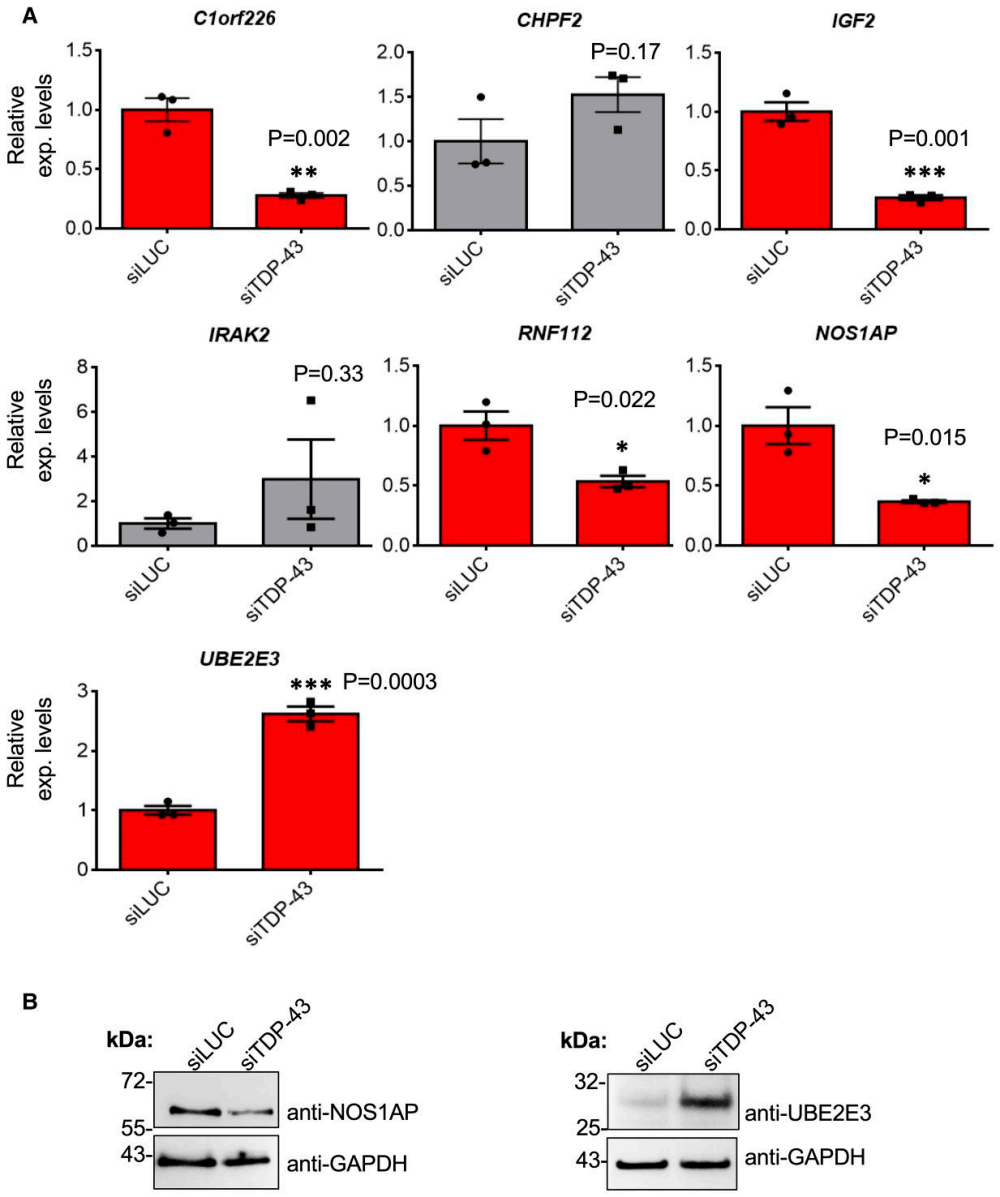
**Analysis of seven co-regulated transcripts among TDP-43 and hnRNPs.** (**A**) qPCR validation of *C1orf226*, *CHPF2*, *IGF2*, *IRAK2*, *RNF112*, *NOS1AP* and *UBE2E3* following TDP-43 knockdown in SH-SY5Y cells. Each bar reports the mean ± SEM of three independent experiments. Nonparametric un-paired *t-test* was considered for statistical significance (*P < 0.05, **P < 0.01, ***P < 0.001). (**B**) Western Blotting analysis of NOS1AP and UBE2E3 expression in siTDP-43 treated cells. Expression of GAPDH is also reported as loading control. For each analysis, three independent experiments were performed, and an exemplificative western blot figure was reported.

Considering that this result was the consequence of a comparison among seven distinct RNA-seq data, we then proceeded with validation using qPCR analysis. As shown in Fig. 4A, the mRNA levels of *C1orf226*, *IGF2*, *RNF112*, *NOS1AP* and *UBE2E3* were all found to be significantly modified (*P* < 0.05) following TDP-43 knockdown. To further confirm these results, we decided to look at protein expression levels of these genes. Particularly, we focused our attention on NOS1AP and UBE2E3 because of their importance in neuronal development^46^ and TDP-43 solubility.^47^ As reported in Fig. 4B, Western Blot analysis of SH-SY5Y cells treated with siTDP-43 versus the control confirmed NOS1AP and UBE2E3 protein down- and upregulation, respectively.

### Mechanistic analysis of NOS1AP regulation by TDP-43

**Figure 5:**
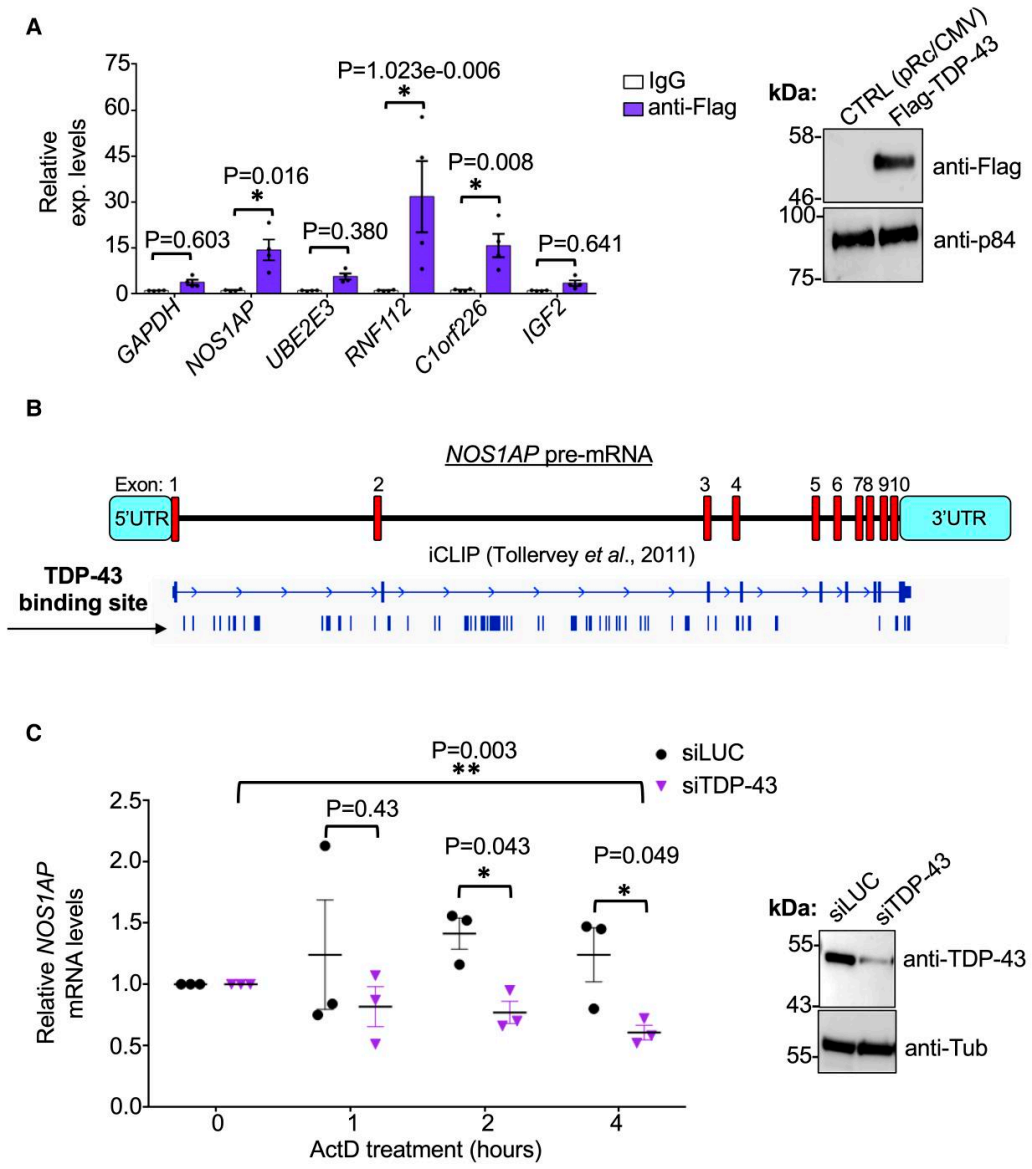
**Characterization of *NOS1AP* and TDP-43 interaction.** (**A**) RNA-immunoprecipitation analysis to check for TDP-43 binding to *NOS1AP*, *UBE2E3*, *RNF112*, *C1orf226* and *IGF2* transcripts and for the housekeeping gene *GAPDH* (used as a control). Each bar reports the mean ± SEM of four independent experiments. Multiple *t-test* was considered for statistical significance (*P < 0.05). Transfection of Flag-tagged TDP-43 was used for the RNA immunoprecipitation assay and the corresponding protein expression was tested by western blotting analysis with respect to the empty vector (pRc/CMV). p84 was used as loading control. (**B**) Schematic representation of *NOS1AP* pre-mRNA (referred to the «canonical isoform» of Uniprot: O75052-1). Exons and regulatory regions are identified in red and blue boxes, respectively. The IGV genome browser’s expanded view of the iCLIP analysis performed by Ule group^48^ is also reported. These data are currently deposited in the ArrayExpress archive and are accessible at E-MTAB-527. iCLIP reads are represented as blue rods along the different gene regions. (**C**) mRNA stability assay of *NOS1AP* transcript following TDP-43 depletion. The relative RNA expression of *NOS1AP* were measured by qPCR at 0, 1, 2 and 4 h after Actinomycin D (Act) treatment (5 µg/ml). *NOS1AP* mRNA levels were normalized against *RPL32*. Values are mean ± SEM of three independent experiments. Nonparametric un-paired *t-test* was considered for statistical significance (*P < 0.05). Protein expression levels of TDP-43 were tested to check the quality of TDP-43 silencing and Tubulin was used as loading control.

To gain further insight into the mechanisms of TDP-43 action on these factors, we first tested for a possible physical interaction between TDP-43 and the various mRNAs. For these experiments, we transfected a Flag-tagged TDP-43 in SH-SY5Y cells and performed immunoprecipitation experiments to check for qPCR enrichment of the various mRNAs compared to control immunoprecipitation using IgG. As shown in Fig. 5A, the mRNAs which resulted directly bound by TDP-43 were *NOS1AP*, *C1orf226* and *RNF112*. No direct binding for TDP-43 could be detected for *UBE2E3*. Interestingly, previously published CLIP data^48^ confirmed that the pre-mRNA of *NOS1AP* contains several TDP-43 binding motifs within its intronic sequences (Fig. 5B), while a lesser degree of interaction was detected for the pre-mRNA of *RNF112*, at the level of intron 1. Regarding the other genes identified by RNA-seq analysis (*UBE2E3*, *IGF2*, *CHPF2*, *IRAK2* and *C1orf226*), we were not able to observe a concordance with our data (qPCR and/or RIP-immunoprecipitation assay) and the putative TDP-43 binding sites detected by CLIP analysis (Supplementary Fig. 1).

In conclusion, NOS1AP represented the best candidate for a functional follow up and this was pursued by testing the stability of the *NOS1AP* mRNA by ActD treatment of SH-SY5Y cells following TDP-43 silencing. Fig. 5C shows that in the absence of TDP-43, the stability of the *NOS1AP* mRNA was significantly impaired, as compared to the normal condition. Considering the high number of potential TDP-43 binding sites we have not been able to identify the ones responsible for this effect of the mRNA stability and further work is currently in progress to clarify this mechanism in detail. Nonetheless, these data are in accordance with the reduction of the *NOS1AP* mRNA levels observed through RNAscope *in situ* hybridization technology following TDP-43 depletion (Supplementary Fig. 2A). Finally, to expand our understanding of TDP-43 and NOS1AP relationship, we evaluated the effects of NOS1AP depletion in SH-SY5Y cells and focused our attention at the localization of TDP-43 and phosphorylated S409/S410 TDP-43. As reported in Supplementary Fig. 2B, we were able to efficiently reduce the protein expression levels of NOS1AP. However, there was no noticeable change in the cellular localization of the endogenous TDP-43 (Supplementary Fig. 3A) and the phosphorylated form of TDP-43, especially in terms of aggregated protein (Supplementary Fig. 3B).

### The *NOS1AP* transcript is downregulated in diseased brain tissues with *STMN2* and *UNC13A* cryptic exons inclusion

**Figure 6:**
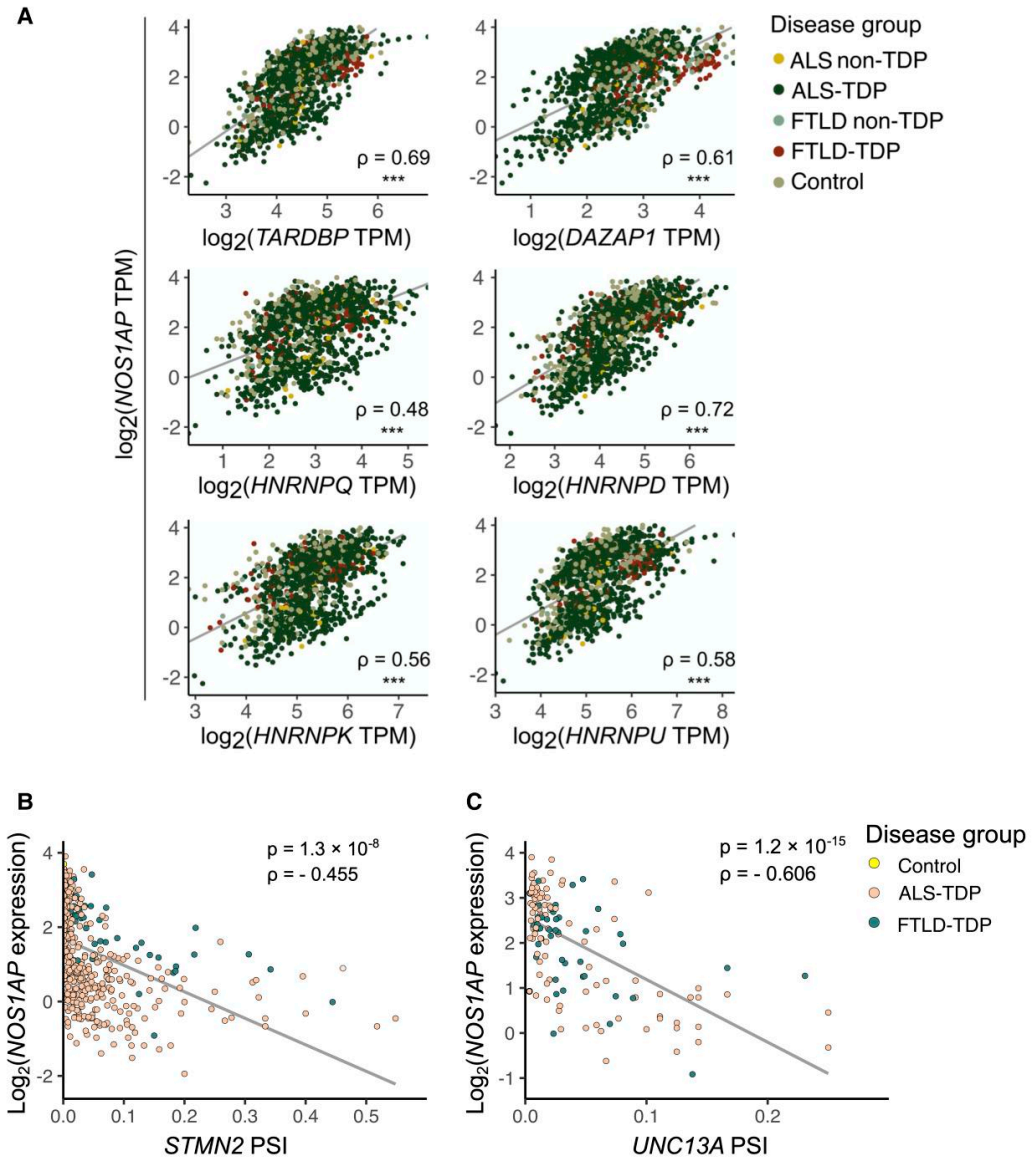
***NOS1AP* expression is reduced in ALS and FTLD patients with TDP-43 pathology.** (**A**) Scatter plots show correlation of *NOS1AP* expression with that of different *hnRNPs* in human samples (*n* = 1349, ALS/FTLD patients or healthy controls) from different brain regions (i.e. cerebellum, cervical spinal cord, frontal cortex, hippocampus, lumbar spinal cord, motor cortex, occipital cortex, temporal cortex and thoracic spinal cord). In this plot, samples from different neuroanatomical regions are plotted together and are not colour-coded, as distinct colours mark brain samples (of any neuroanatomical region) derived from patients diagnosed with a certain disease or not. Gene expression is plotted as unadjusted log_2_-transformed TPM values, Spearman’s ρ is shown on each plot, *** marks unadjusted *P* < 2.2 × 10^−16^. Moreover, *NOS1AP* expression plotted as log_2_-transformed TPM values negatively correlates with inclusion levels (PSI) of cryptic exons within (**B**) *STMN2* (*n* = 455, Spearman’s ρ = −0.46, *P* = 1.3 × 10^−8^) and (**C**) *UNC13A* transcript (*n* = 142, Spearman’s ρ = −0.61, *P* = 1.2 × 10^−15^) in different neuroanatomical regions (i.e. cerebellum, cervical spinal cord, frontal cortex, hippocampus, lumbar spinal cord, motor cortex, occipital cortex, temporal cortex and thoracic spinal cord) of ALS and FTLD patients with reported TDP-43 pathology. Only samples with detected cryptic inclusion (PSI > 0) were considered herein. Samples of ALS-TDP patients are shown in orange and those of FTLD-TDP in brown. There is one case of a healthy individual (control) with cryptic inclusion within *STMN2* that is shown in yellow. Grey lines represent fitted regression.

As *NOS1AP* appears to be under TDP-43’s control as well as under control of other members of the hnRNP family, we investigated possible correlation of *NOS1AP* levels with all the six hnRNPs analysed in our work. To do this, we took advantage of a large RNA-seq cohort of human brain samples (the NYGC ALS cohort). In this big data set, we detected significant associations between abundance level of *NOS1AP* and that of *TARDBP* and of the modifier hnRNPs. These data suggest that mRNA levels of *NOS1AP* are subject to hnRNP control also *in vivo* and not just in our SH-SY5Y cell line (Fig. 6A). It should be noted, however, that this evidence is largely correlative, leaving causal relationships to be established experimentally.

Most importantly, we also wished to explore whether *NOS1AP* expression potentially correlated with TDP-43 dysfunction *in vivo*. As TDP-43 dysfunction cannot be measured in a direct fashion, inclusion of cryptic exons within Stathmin-2 (*STMN2*) and Unc-13 homolog A (*UNC13A*) transcripts has recently been proposed as representing a proxy of TDP-43 pathology.^37,38^ Looking at *NOS1AP* levels across various brain regions of patients with reported TDP-43 pathology, in which we could additionally detect cryptic exon inclusion (PSI > 0, Fig. 6B and C), we indeed observed reduced *NOS1AP* levels in tissues with higher cryptic exon burden (Fig. 6B for *STMN2* and Fig. 6C for *UNC13A*, respectively), which presumably points towards more severe disease phenotype.

### Functional importance of TDP-43 induced NOS1AP downregulation in primary cortical cultures

As NOS1AP, through direct or indirect interaction with important synaptic proteins, is involved in physiological and pathophysiological processes (such as dendrites development and maintenance, neurotransmission and neurotoxicity^46,49,50^), we sought to investigate the effect of TDP-43 downregulation on *Nos1ap* expression levels in rodent cortical cultures. To this aim, mouse primary cortical cells were plated and transfected at DIV 5 with a pZac2.1-GFPsh-mTDP-43 vector, which allowed fluorescence-activated cell sorting sorting of transfected versus nontransfected cells from the same culture dish (Supplementary Fig. 4).

**Figure 7:**
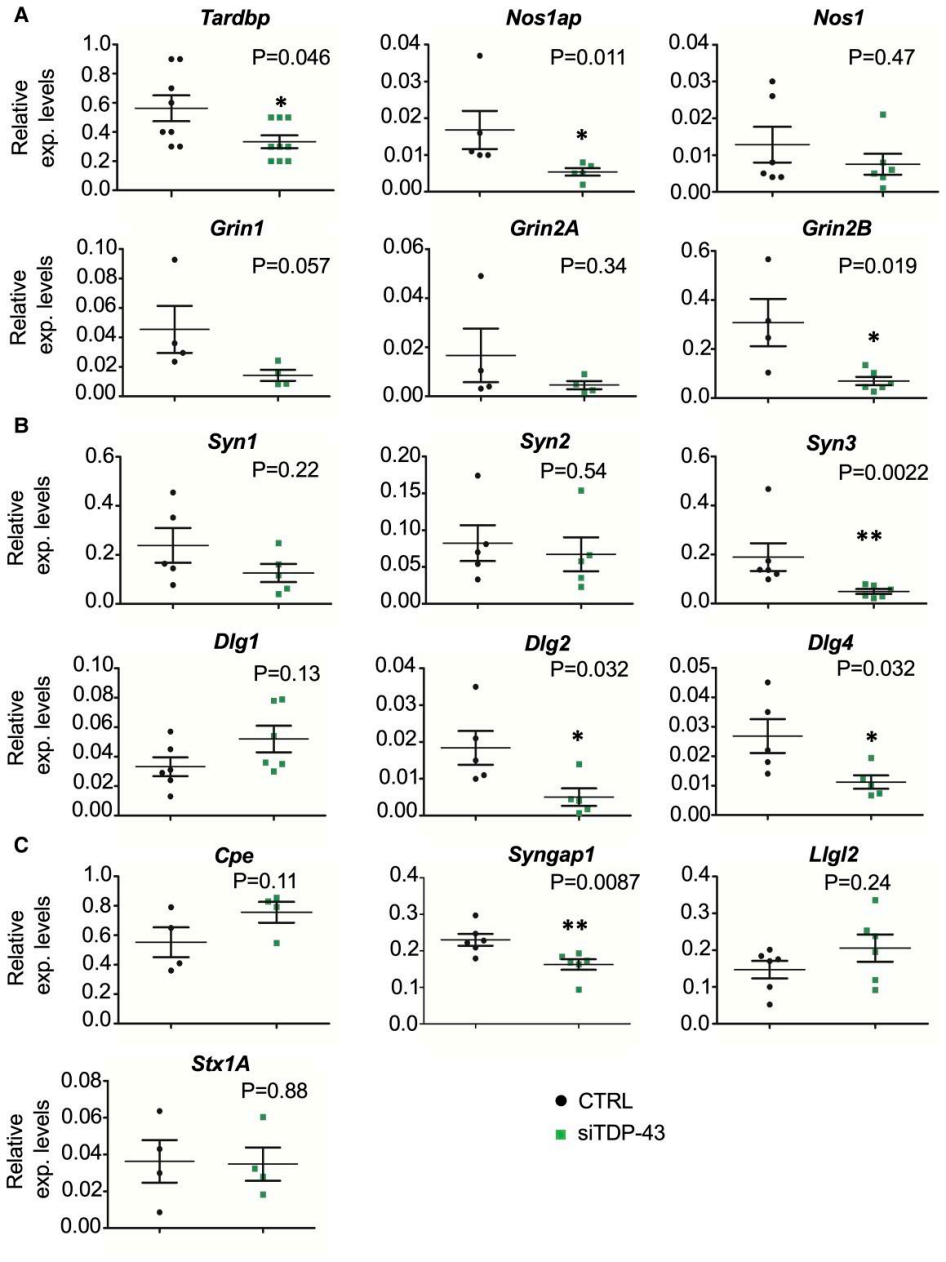
**TDP-43 silencing in cortical cultures. Neuronal cultures were transfected with the pZac2.1-GFPsh-mTDP-43.** After sorting and mRNA extraction, *Tardbp* (mouse TDP-43) and *Nos1ap* mRNAs levels were measured in the sorted control cells (CTRL, not transfected GFP negative) and siTDP-43 transfected (GFP positive) cells. (**A**) Quantitative real-time PCR analysis showed a significant decrease of the *Tardbp*, *Nos1ap* and *Grin2b* (coding for GluN2B) mRNAs in transfected cells compared to control cells (CTRL), while the mRNA expression of *Nos1*, *Grin1* (coding for GluN1), and *Grin2a* (GluN2A) did not change. (**B and C**) The mRNA expression of Synapsin-3 (*Syn3*), *Dlg2* (coding for PSD93), *Dlg4* (PSD95) and *Syngap1* was also significantly decreased. Synapsin-1 (*Syn1*) and -2 (*Syn2*), Carboxypeptidase E (*Cpe*), Scribble component 2 (*Llgl2*), Syntaxin-1 (*Stx1A*) and SAP97 (*Dlg1*) mRNAs did not change. Values were normalized to the levels of the housekeeping gene *RPL34* from four independent experiments. Error bars indicate mean ± SEM. Statistically significantly differences are indicated as follows: *P < 0.05; **P < 0.01 using the Mann–Whitney test.

On the one hand, we observed a substantial drop of *Tardbp* (TDP-43) mRNA expression following transient transfection of the specific shRNA. On the other hand, this reduction was associated with a significant decrease in *Nos1ap* mRNA and a minor (though not significant) reduction of Neuronal nitric oxide synthase (*nNos/Nos1*) mRNA (Fig. 7A). Importantly, the *Nos1ap* mRNA decrease was correlated with a significant drop in the mRNA of some of the predicted NOS1AP interacting factors, specifically *Grin2B* encoding for the GluN2B subunit of the NMDAR (Fig. 7A), as well as *Dlg2* (PSD93/Chapsyn110) and *Dlg4* (PSD95/SAP90), members of the MAGUKs family and components of the post-synaptic formation (Fig. 7B). Conversely, the expression levels of *Dlg1* mRNA encoding for SAP97, a fellow MAGUK member, and not a known NOS1AP networking protein, were not significantly modified (Fig. 7B). Since Synapsins are a family of neuron-specific phosphoproteins implicated in synaptogenesis and neurotransmitter release^51,52^ and they are known binding partners of NOS1AP,^53^ we asked whether the TDP-43/NOS1AP downregulation also affects the mRNA expression of these presynaptic proteins. Our analyses showed a significant decrease in the mRNA encoding for Synapsin-3 (*Syn3*) (Fig. 7B). To get deeper insight into the effects of the TARDBP/NOS1AP downregulation at the Postsynaptic density (PSD), we analysed the Synaptic GTPase-Activating protein (SynGAP), a key PSD synaptic protein linked to postsynaptic scaffold proteins (PSD93 and PSD95) and the NMDAR.^54,55^ As compared to nontransfected cells (CTRL), we observed a significant decrease in *Syngap1* mRNA (Fig. 7C). The downregulation of TDP-43/NOS1AP had no effect on the mRNA expression of Syntaxin-1 (*Stx1A*) (Fig. 7C). Syntaxin-1 is a component of the *N*-ethylmaleimide-sensitive factor attachment protein receptor (SNARE) complex, essential for neurotransmission, although it is not a known member of the NOS1AP network. We further assessed the mRNA expression of other proteins known to interact with NOS1AP/CAPON: Carboxypeptidase E (*Cpe*) and Scribble complex (*Llgl2*). They are involved in dendrite morphology,^50,56^ cellular polarity and synaptogenesis,^57^ and we found that were not altered (Fig. 7C).

**Figure 8:**
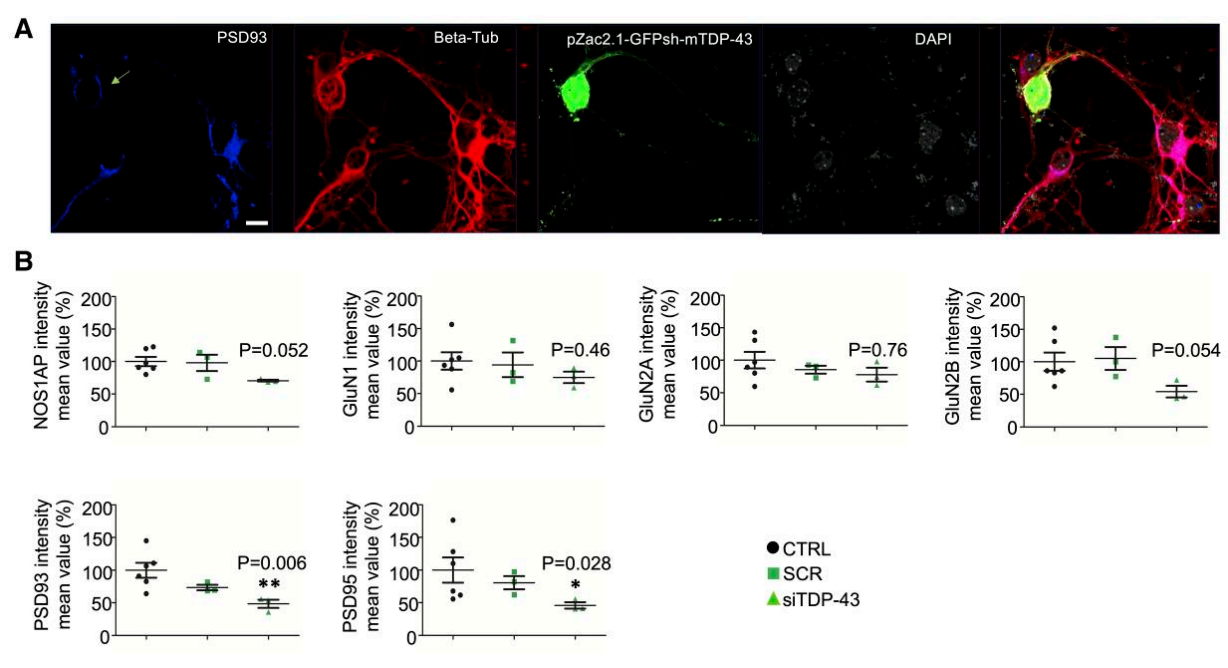
**TDP-43 silencing in cortical neurons alters the expression of selected post-synaptic proteins.** (**A**) Representative immunofluorescence of primary cortical neurons labelled with PSD93 (blue), beta-tubulin (red), the pZac2.1-GFPsh-mTDP-43 plasmid vector (green), and DAPI (grey). Beta-tubulin was used to identify neurons. (**B**) Measurement of the mean intensity value of NOS1AP, GluN1, GluN2A and GluN2B, PSD93 and PSD95, proteins in neuronal perisomatic regions, respectively, of siTDP-43 transfected cells compared to nontransfected cells (CTRL). Downregulation of PSD93, and PSD95 and a near significance of NOS1AP and GluN2B proteins, in the perisomatic region, were observed in siTDP43 transfected cells versus CTRL. The graphs show the mean data as percentage normalized to CTRL values obtained from three independent cell experiments from a minimum of three different fields. Error bars indicate mean ± SEM. Statistically significant differences between CTRL and siTDP-43 conditions are indicated as follows: *P < 0.05, ** *P* < 0.001 using the Kruskal–Wallis test. The proteins expression in the scramble (SCR) transfected cells was not significant different compared to CTRL and siTDP-43 neurons. Scale bar: 10 μm.

As all these changes were detected at the mRNA level, we then examined whether the TDP-43/NOS1AP downregulation also affects the protein expression of PSD93/Chapsyn-110 and PSD95/SAP90 and of the GluN1, GluN2A and GluN2B NMDAR subunits. Quantitative fluorescence microscopy assays that measure relative changes in the level of the protein of interest were carried out on the somata and perisomata regions of beta-tubulin+ cells (see Material and Methods and Supplementary Fig. 5A and B). The quantitative analyses of the neuronal somata immunostaining did not reach statistical significance (Supplementary Fig. 6B), while the analyses of the perisomata region showed a decrease in the transfected neurons (siTDP-43) of the immunofluorescence intensity of NOS1AP, GluN2B, PSD93/Chapsyn-110 and PSD95/SAP90 (Fig. 8B) proteins.

### NOS1AP can functionally rescue TDP-43 induced toxicity in *Drosophila* and TDP-43 controlled events in human SH-SY5Y cells

**Figure 9:**
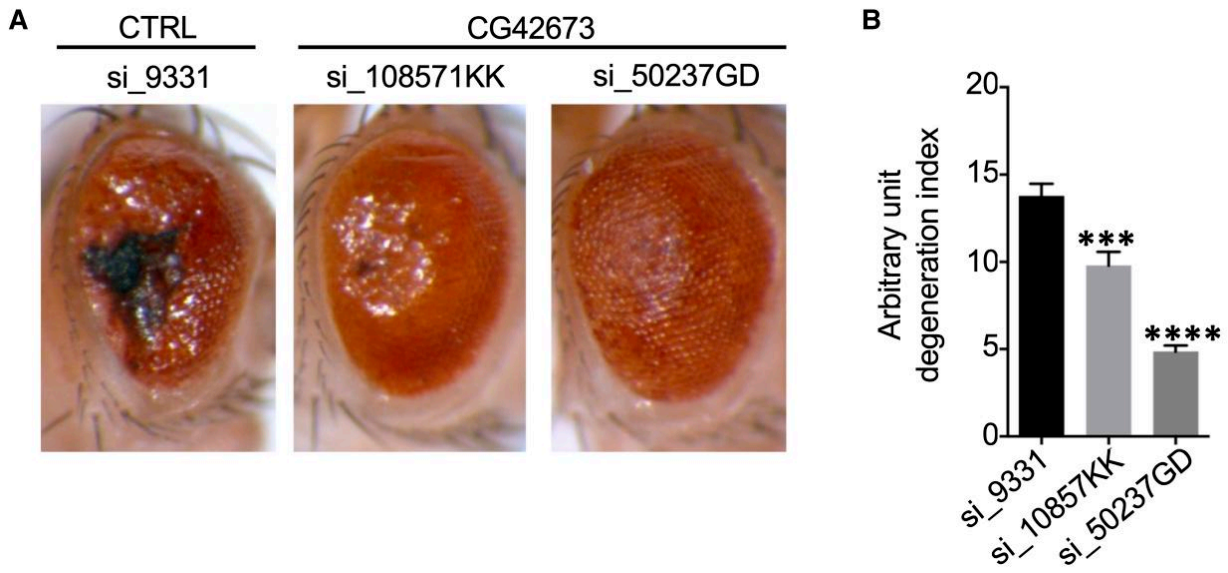
**Contribution of NOS1AP downregulation in the regulation of fly TDP-43 (TBPH)-induced toxicity.** (**A**) Eye phenotype of flies expressing UAS TBPH and siRNA for CG42673. Expression of TBPH-induced degeneration in Drosophila eye and the degenerative phenotype were rescued by the co-expression of siRNA silencing gene CG42678: control with unrelated siRNA against GFP: GMR-Gal4, UAS-TBPH/si_9331, siRNA for CG42673 VDRC KK library: GMR-Gal4, UAS-TBPH/si_108571, siRNA for CG42673 VDRC GD library: si_50237GD; GMR-Gal4, UAS-TBPH. (**B**) Eye phenotype quantification: arbitrary units of eye degeneration index. The co-expression of both siRNA silencing CG42673 rescued TBPH-induced eye degeneration. Statistical analysis was performed with Prism (GraphPad) version 6.0.: si_9331 (*n* = 52), si_108571 (*n* = 24), si_50237 (*n* = 40). One-way ANOVA with Bonferroni correction was applied as statistical test. Values were displayed as mean ± SEM. Statistical significance displayed as: ***P < 0.001, ****P < 0.0001.

Finally, as we first identified these hnRNPs in a *Drosophila* model of TDP-43 pathology, we asked whether fly homologues of the factors identified in this study (Fig. 3B, lower Venn diagram) had any ability to rescue TDP-43 pathology on their own. Of the seven factors reported in Fig. 3B, the two which had the greatest homology with *Drosophila* proteins were NOS1AP and UBE2E3. Thus, we found that targeted RNAi knockdown of both CG42673 (ortholog of the human NOS1AP) and CG6720 (ortholog of the human UBE2E3) in the eyes reduced the ocular degeneration induced by UAs-TBPH (fly homologue of the human TDP-43) overexpression. In the case of CG42673, both UAS si_108571KK and UAS si_50237GD caused a statistically significant improvement of eye morphology versus an unrelated RNAi against GFP (Fig. 9A and B). Interestingly, also the knockdown of CG6720 (UAS si_31158GD) in the eyes of flies expressing UAS-TBPH was associated with a reduced degeneration and amelioration of eye morphology, compared with an unrelated control RNAi against GFP (Supplementary Fig. 9A and B). In the future, we plan to use this information to accurately dissect the importance of the various isoforms and protein domains of NOS1AP that are responsible for this rescue-effect in flies.

**Figure 10:**
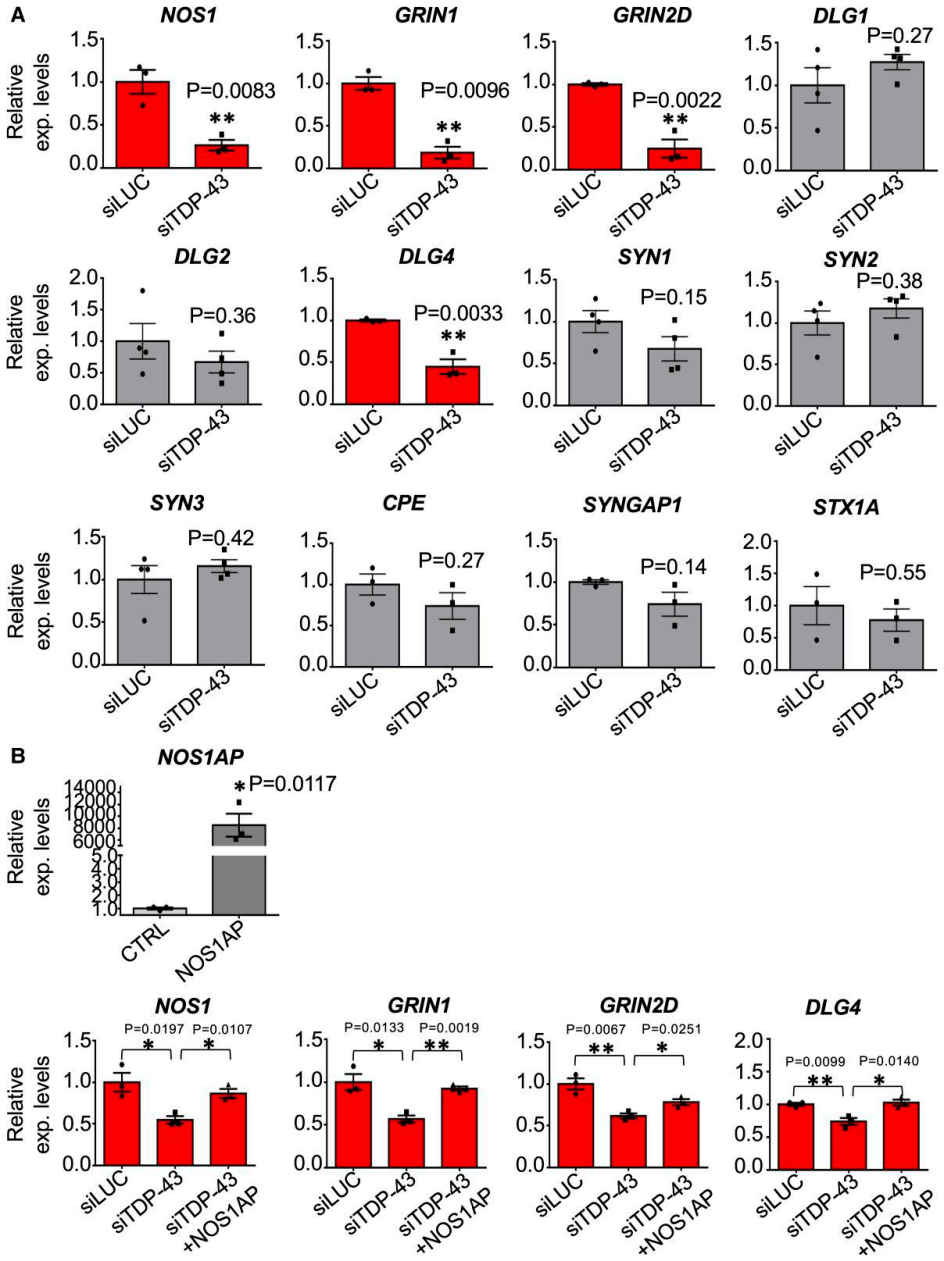
**Contribution of TDP-43 mediated NOS1AP depletion in regulating the NMDA receptor signalling in human SH-SY5Y cells** (**A**) qPCR analysis of NMDAR-related genes following TDP-43 knockdown in SH-SY5Y cells. Each bar reports the mean ± SEM of three independent experiments (for *NOS1*, *GRIN1*, *GRIN2D*, *DLG4*, *CPE*, *SYNGAP1* and *STX1A* genes) or four independent experiments (for *DLG1*, *DLG2*, *SYN1*, *SYN2*, *SYN3* genes). Nonparametric un-paired *t-test* was considered for statistical significance (**P < 0.01). (**B**) Effects of TDP-43 downregulation on *NOS1*, *GRIN1*, *GRIN2D* and *DLG4* mRNA levels comparing to control (siLUC treated cells) following overexpression of NOS1AP in siTDP-43 depleted cells. *NOS1AP* overexpression was also controlled by qPCR and is reported in the figure. Each bar reports the mean ± SEM of three independent experiments. Nonparametric un-paired *t-test* was considered for statistical significance (*P < 0.05, **P < 0.01).

Finally, to further clarify the role of NOS1AP in TDP-43 pathology, we decided to focus our attention on the NMDA receptor pathway in human SH-SY5Y cells. We first assessed the ability of TDP-43 to control the corresponding human genes belonging to the NMDA signalling described in mouse cortical neurons. To achieve this, we silenced TDP-43 and evaluated the mRNA expression of *NOS1*; two NMDAR subunits strongly expressed in this human cell line, *GRIN1* (encoding GluN1) and GRIN2D (encoding GluN2D); the MAGUK genes *DLG1*, *DLG2*, *DLG4*; the synapsin members *SYN1, SYN2*, *SYN3* as well as other NOS1AP interactors such as *CPE*, *SYNGAP1* and *STX1A* transcripts. Notably, we found a significant reduction of *NOS1*, *GRIN1*, *GRIN2D* and *DLG4* mRNAs after siTDP-43 treatment (Fig. 10A), indicating that, in humans, TDP-43 can act on the same neuronal pathways apparently affected in mouse cells. Most importantly, to further support that downregulation of these TDP-43 controlled events was mediated by the NOS1AP depletion, we also carried out overexpression of NOS1AP in siTDP-43 treated cells and assessed the eventual recovery of expression level of each gene, at the mRNA level. Our results are reported in Fig. 10B and clearly show the rescue of levels of *NOS1*, *GRIN1*, *GRIN2D* and *DLG4* transcripts.

## Discussion

In the present study, we have performed transcriptome analysis of SH-SY5Y cells silenced for *DAZAP1*, *hnRNP-Q*, *hnRNP-D*, *hnRNP-K* and *hnRNP-U* that were known to affect TDP-43 pathology. The study was performed in order to investigate the connection of these hnRNPs with TDP-43, one of the most relevant proteins involved in ALS and FTLD.^8^

After cross-comparing transcriptomic profiles of cells depleted by each of these factors, we identified seven commonly regulated transcripts: *CHPF2*, *IGF2*, *IRAK2*, *RNF112*, *UBE2E3*, *C1orf226* and *NOS1AP*. Interestingly, multiple lines of evidence have linked these genes to neuronal functions and, in several cases, already in association with TDP-43 pathology. In particular, *CHPF2* gene encodes a chondroitin glucuronyltransferase that has been reported by Malacards database^58^ to be associated with a rare genetic form of mental retardation, namely Coffin Siris Syndrome. In 2016, Allodi and collaborators have demonstrated that IGF2 was capable to prevent ALS-like toxicity in human spinal motor neuron.^44^ Although no functional connection has already been described, *IRAK2* encodes a putative serine/threonine kinase that has been described as a TDP-43 interacting protein via affinity capture-mass spectrometry assay.^59^ RNF112, also known as Znf179, is a zinc-finger protein abundant in the nervous system that is involved in neuroprotection against superoxide radicals^60^ and in neuronal differentiation.^61^ Interestingly, Znf179 shows an ubiquitin ligase activity and it has been found to induce the polyubiquitination of TDP-43 in mouse brain reducing its insoluble aggregates.^62^ Likewise, UBE2E3 has been shown to participate in the regulation of the oxidative stress^63^ and it is an ubiquitin-conjugating enzyme that participates in controlling the TDP-43 neurotoxicity.^47^ Finally, C1orf226 is an uncharacterized gene and its sequence follows the *NOS1AP* gene on chromosome 1.

Out of our list, NOS1AP (also known as CAPON) has recently emerged as an important player in brain physiology and pathophysiology. Several studies suggest that its interaction with nNOS contributes to NOS1AP-mediated excitotoxicity, the formation of neuronal processes and probably schizophrenia.^50,64,65^ More recently, Li *et al.* have reported a NOS1AP-regulated neuronal cell death downstream of the NMDAR^66^ and Hashimoto *et al.* have identified NOS1AP as a tau-binding protein.^67^

In our cross-comparison, *NOS1AP* represents the most interesting transcript identified by our RNA-seq analysis, its RNA being a direct binding target of TDP-43 and its downregulation is capable to rescue on its own the degenerative phenotype induced by TDP-43 overexpression in fly eyes. Most importantly, we also observed a clear correlation between the reduction of NOS1AP and the inclusion of two previously characterized cryptic exons in different brain regions of patients with TDP-43 pathology. Overall, these observations support the hypothesis of an important involvement of NOS1AP in TDP-43 pathological pathways.

Furthermore, using primary mouse cortical cultures, we have also demonstrated that the concurrent decrease of TDP-43 and NOS1AP elicits a significant downregulation at the mRNA level of several factors that directly or indirectly interact with NOS1AP.^64,68^ Among others, we found components of the post-synaptic density (PSD) and of the NMDARs that could represent an important event in the pathology, considering their critical role in numerous types of plasticity.^69,70^ Structurally, NMDARs are hetero-tetramers form by a mandatory GluN1 subunit, with combinations of GluN2/GluN3 subunits that modulate channel properties.^71^ In our study, we found a significant decrease in three essentials component of the PSD: PSD93 and PSD95, two members of the MAGUKs family of scaffolding proteins, and of SynGAP, a key PSD signalling enzyme physically linked to PSD95.^72,73^ The PDZ (PSD95–DLG1–ZO1) domains of PSD93 and PSD95 directly associates with the PBMs (PDZ-binding motifs) at the C-terminal cytoplasmic tail of the NMDAR subunits.^74^ These interactions are crucial for the trafficking, clustering and removal of the receptor at the synapse.^75,76^ Our finding of a concurrent decreased of the mRNA and proteins of PSD93, PSD95 and the NMDAR subunit GluN2B is consistent with the tight interactions between these proteins.^77^ Furthermore, Frank *et al.*^78^ reported the indispensable presence of both PSD93 and PSD95 for the formation of NMDAR complexes and the importance of the GluN2B subunit for the assembly of the NMDA/PSD93-PSD95 complexes. An additional key molecule highly enriched at excitatory synapses^79^ and closely associated with NMDARs through the scaffolding proteins of the PSD is SynGAP.^80^ We found a significant decrease in *SynGAP* mRNA further underscoring how the concomitant decrease of TDP-43 and NOS1AP has striking effects on the PSD compartment.

In agreement with previous studies identifying synapsins as binding partners of NOS1AP,^53^ here we also report a significant decrease of the mRNA encoding for *Synapsin-3* in the siTDP43 cortical cultures. Within the Synapsin family, Synapsin-3 holds some peculiar features: its activity (contrarily to Synapsin-1 and -2) is inhibited by Ca^2+^ at physiological concentrations; it is involved in axonal elongation and growth-cone formation; it enhances the probability of GABA to be released from the readily releasable pool (RRP) and regulates the size of the RRP.^81-83^ A recent study demonstrated that mice lacking Synapsin-3 exhibited a diminished behavioural flexibility, in other words a diminished ability to modify a behaviour in a changing environment.^84^ In this respect, cognitive inflexibility and apathy-like behaviour are features described in ALS–FTD patients.^85^

It is intriguing that we have observed meaningful changes in the mRNA expression of component of the PSD linked to the NMDAR and of Synapsin-3 linked to the release of GABA, since emerging evidence link the FTD neuropathology with general alteration in several neurotransmitter systems including the glutamatergic and GABAergic systems.^86-88^

Some limitations of this study should also be mentioned. Although our analyses focused on member of the pre- and post-synaptic compartments, our sorting protocol did not separate neuronal from non-neuronal cells, and thus we cannot completely rule out a possible contamination of non-neuronal cells in the mRNA analyses. Yet, the immunohistochemistry data, performed on beta-tubulin positive cells are consistent with the mRNA analyses, suggesting that altogether our data could accurately reflect the impact of the TDP-43/NOS1AP downregulation on the neuronal pre- and post-synaptic compartments. Furthermore, in the mouse cortical cultures, we cannot rule out the fact that the downregulation of NOS1AP could directly affect the transcription level (mRNA) of some of its proteins partners, or that this decrease could be the result of TDP-43 decrease independently of NOS1AP. However, rescue experiments performed in human SH-SY5Y cells suggest that there is a significant dependence of the NMDAR pathway on the TDP-43-NOS1AP balance mediated by different genes, such as *NOS1*, *GRIN1*, *GRIN2D* and *DLG4*.

Nonetheless, taken together all the evidence, we believe that our identification of NOS1AP as a co-regulated target by several hnRNP proteins, including TDP-43, and the role of NOS1AP in the synaptic signalling can link this gene to neurological dysfunctions associated with TDP-43 pathology, make this gene a suitable candidate for the development of novel therapeutic strategies in the context of ALS–FTD pathology.

## Supplementary Material

fcac242_Supplementary_Data

## Appendix

List of the NYGC ALS Consortium members: Hemali Phatnani, Justin Kwan, Dhruv Sareen, James R. Broach, Zachary Simmons, Ximena Arcila-Londono, Edward B. Lee, MD, Vivianna M. Van Deerlin, MD, Neil A. Shneider, Ernest Fraenkel, Lyle W. Ostrow, Frank Baas, Noah Zaitlen, James D. Berry, Andrea Malaspina, Pietro Fratta, Gregory A. Cox, Leslie M. Thompson, Steve Finkbeiner, Efthimios Dardiotis, Timothy M. Miller, Siddharthan Chandran, Suvankar Pal, Eran Hornstein, Daniel J. MacGowan, Terry Heiman-Patterson, Molly G. Hammell, Nikolaos. A. Patsopoulos, Oleg Butovsky, Joshua Dubnau, Avindra Nath, Robert Bowser, Matt Harms, Eleonora Aronica, MD, Mary Poss, Jennifer Phillips-Cremins, John Crary, Nazem Atassi, Dale J. Lange, Darius J. Adams, Leonidas Stefanis, Marc Gotkine, Robert H. Baloh, Suma Babu, MBBS, Towfique Raj, Sabrina Paganoni, Ophir Shalem, Colin Smith, Bin Zhang, Brent Harris, Iris Broce, Vivian Drory, John Ravits, Corey McMillan, Vilas Menon. See Supplementary Table 3 for the author affiliation.

## Abbreviations

ActD =: actinomycin D
ALS =: amyotrophic lateral sclerosis
APOA2 =: apolipoprotein A2
*C1orf226* =: Chromosome 1 Open Reading Frame 226
*CHPF2* =: Chondroitin Polymerizing Factor 2
Cpe =: Carboxypeptidase E
DAZAP1 =: deleted in azoospermia-associated protein 1
DEGs =: differentially expressed genes
DIV =: Days *in vitro*
EWSR1 =: EWS RNA-binding protein
fALS =: familial amyotrophic lateral sclerosis
FC =: Fold Change
FTD =: frontotemporal dementia
FTLD =: frontotemporal lobar degeneration
FUS =: fused in sarcoma
FXTAS =: Fragile X-associated Tremor/Ataxia Syndrome
GAPDH =: Glyceraldehyde-3-phosphate dehydrogenase
GluN1 =: NMDA receptor subunit 1
GluN2A =: NMDA receptor subunit 2A
GluN2B =: NMDA receptor subunit 2B
hnRNPs =: heterogeneous nuclear ribonucleoproteins
IGF2 =: Insulin-Like Growth Factor 2
*IRAK2* =: Interleukin 1 Receptor-Associated Kinase 2
LCD =: low complexity sequence domain
Llgl2 =: Scribble Cell Polarity Complex Component 2
LLPS =: liquid–liquid phase separations
MAGUK =: membrane-associated guanylate kinases
MATR3 =: Matrin-3
NMDAR =: *N*-methyl-D-aspartate receptor
NOS1 =: neuronal nitric oxide synthase
NOS1AP =: Nitric Oxide Synthase 1 Adaptor Protein
POLR2A =: RNA polymerase II subunit A
pre-mRNA =: pre-messenger RNA
PSD =: post-synaptic density
PSD93 =: Postsynaptic Density Protein 93 (or channel-associated protein of synapse-110)
PSD95 =: Postsynaptic density protein 95 (or synapse-associated protein 90)
qPCR =: quantitative PCR
RNA-IP =: RNA-immunoprecipitation
*RNF112* =: Ring Finger Protein 112
RPL13A =: Ribosomal Protein L13a
RPL32 =: Ribosomal Protein L32
RPL34 =: Ribosomal Protein L34
RRP =: readily releasable pool
sALS =: sporadic amyotrophic lateral sclerosis
SNARE =: *N*-ethylmaleimide-sensitive factor attachment protein receptor
SORT1 =: Sortilin 1
STMN2 =: Stathmin-2
Stx1A =: Syntaxin-1
Syn-3 =: Synapsin-3
Syn1 =: Synapsin-1
Syn2 =: Synapsin-2
SynGAP =: Synaptic GTPase-Activating protein
TAF15 =: TATA-binding protein-associated factor 2N
TDP-43 =: TAR DNA-binding protein 43 kDa
*UBE2E3* =: Ubiquitin-Conjugating Enzyme E2 E3
UNC13A =: Unc-13 homolog A
