# Supplementary material for: NOS1AP is a novel molecular target and critical factor in TDP-43 pathology
Source: Brain Commun. 2022 Sep 23;4(5):fcac242. doi: 10.1093/braincomms/fcac242 (PMC9576154; doi:10.1093/braincomms/fcac242)
Supplement: fcac242_Supplementary_Data [file fcac242_supplementary_data.zip › Supplementary_material_and_uncropped_blots.docx]

**Supplementary Material**

**Human neuroblastoma SH-SY5Y cell culture**

Human neuroblastoma SH-SY5Y cell line (ECACC) was cultured up to 20 passages in Dulbecco’s modified Eagle’s medium (DMEM)/Nutrient Mixture F-12 Ham (Sigma-Aldrich), supplemented with 15% fetal bovine serum (FBS) (Sigma-Aldrich), 1% MEM Non-essential Amino Acid Solution (100X) (Sigma-Aldrich), and 1% Antibiotic-Antimycotic-stabilized suspension (Sigma-Aldrich) at 37°C with humidified atmosphere of 5% CO2.

**NOS1AP Knockdown in SH-SY5Y cells**

Regarding the silencing of *NOS1AP* and its relative control siRNA, we used pre-designed ON-TARGET plus Human *NOS1AP* siRNA-SMART pool (Dharmacon) and ON-TARGET plus Non-targeting Pool (Dharmacon), respectively. Lipofectamine 3000 transfection reagent (Invitrogen) was used to perform two rounds of silencing. Briefly, 45x10^4^ cells were seeded in 6-well plates (at day 0) and silenced in forward transfection with a mixture of 125 µl Opti-MEM (Life-Technologies), 1 µl of 40 µM siNOS1AP or control siRNA (siNEG) and 7.5µl of Lipofectamine 3000 reagent. The final siRNA concentration in each plate was 27 nM and two consequent rounds of silencing were performed at day 1 and day 2. After 72 hours from the second silencing (day 5), cells were collected and prepared for western blot or used for immunofluorescence analysis.

**Fly strains and maintenance**

The genotype of the flies used in this work are indicated below:

w;GMR-Gal4,UAS-TBPH

si_9331: UAS-siRNAGFP (#9331 Bloomington)

si_108571KK: UAS-siRNA CG42673 (#108571, VDRC KK-library)

si_50237GD: UAS-siRNA CG42673 (#50237, VDRC GD-library)

si_31158GD: UAS-siRNA CG6720 (#31158, VDRC GD-library)

All flies were maintained at 25°C, with a 12:12 hour light:dark cycle, on standard cornmeal food (agar 6.25 g/L, yeast 62.5g/L, sugar 41.6 g/L, flour 29 g/L, propionic acid 4.1ml/L).

**Animal care and cortical cells cultures**

Cortical primary cells cultures were obtained from the C57BL/6 mice. Before the sacrifice mice were housed at the Santa Lucia Foundation animal facility. Animal care and use followed the European Directive (2010/63/EU) adopted by the Council of the European Union for animal experiments and adequate measures were taken to minimize pain or discomfort. The experimental protocol was approved by the Italian Ministry of Health (license Number 424/2019-PR). Mixed cortical cultures were prepared as described^1^ from E15-day-old-embryos (hence called E15). Brains were removed from the skull, freed of meninges, and cortical hemispheres dissected from the rest of the brain. After dissection, incubation in 0.025% trypsin and gentle trituration with a fire polished Pasteur pipet, cells were plated at a constant density of 5x10^4^ cells in 2 ml of medium (Neurobasal, 5% FBS, 2% B27, 1mM Glutamine, and 100 IU/ml penicillin/100 μg/ml streptomycin), on poly-L-lysine–coated petri dishes [35 mm]. After 3 days the medium was replaced with Neurobasal, 2% B27, 300mM L-glutamine and 100 IU/ml penicillin/100 μg/ml streptomycin.

**Primary antibodies for Western Blot analysis**

Protein expression analysis was performed using the primary antibodies: rabbit polyclonal anti-TDP-43 (1:1000, Proteintech 10782-AP), rabbit polyclonal anti-hnRNP-D (1:500, Sigma-Aldrich HPA004911), rabbit polyclonal anti-hnRNP-K antibody (1:250, Sigma-Aldrich HPA044105), rabbit polyclonal anti-hnRNP-U (1:250, Sigma-Aldrich HPA058707), rabbit monoclonal anti-CAPON/NOS1AP (1:500, Abcam ab190686), mouse monoclonal anti-UBE2E3 (1:1000, Origene OTI4C8) or mouse monoclonal anti-FLAGM2 (1:1000, Sigma-Aldrich F1804). Mouse polyclonal anti-Tubulin (1:10000, homemade), mouse monoclonal anti-GAPDH (1:1000, Invitrogen MA5-15738) or mouse monoclonal anti-p84 (1:1000 Abcam ab487) were used as protein loading controls.

**List of qPCR primers**

**Supplementary Table 4.** List of primers using for Real-Time PCR of human SH-SY5Y cells

| **Gene (PROTEIN)** | **Sequence (5’-3’)** |
| --- | --- |
| *TARDBP (*TDP-43*)* | F-atctggtgtatgttgtcaactatcc R-gaacttctccaaaggtactaaaatactc |
| *HNRNPD* (hnRNP-D) | F-gcgtgggttctgctttattacc R-ttgctgatattgttccttcgaca |
| *HNRNPK* (hnRNP-K) | F-gcaggaggaattattggggtc R-tgcactctacaaccctatcgg |
| *HNRNPU* (hnRNP-U) | F-gagcatcctatggtgtgtcaaa R-tgaccagccaatacgaacttc |
| *C1orf226* | F-cctcccataaccagaaagcga R-tctgtcccatactccagactg |
| *CHPF2* | F-cttcccctcatcttagggctg R-catcactttggtctagccgag |
| *IGF2* | F-ccgtgcttccggacaactt R-ctgcttccaggtgtcatattgg |
| *IRAK2* | F-gaaatcaggtgtcccattccag R-tggggaggtcgcttctcaa |
| *RNF112* | F-tgagcagggaaacaaggatc R-ggatgagtcacgaactaagaggt |
| *NOS1AP* | F-agccaagaacatcaagaagaag R-tcactttcactccatccactg |
| *UBE2E3* | F-tggagtcccgctttgactatt R-atcctgtcgtgttctgctct |
| *CPE* | F-cttggcccagtacctatgcaa R-accagtccttgagttcaccag |
| *DLG1* | F-tactccccagtttctaaagcagt R-caggtcctccggctaagataa |
| *GRIN1* | F-ctaccgcatacccgtgctg R-gcatcatctcaaaccacacgc |
| *GRIN2D* | F-gtgggataaccgggattactcc R-gaagcgaccatagcgggac |
| *NOS1* | F-ctgttaaccatgtcaagtagcca R-gttccagactcggaagtcgtg |
| *DLG2 (PSD93)* | F-gccggtgattatcctgggg R-cgctttggcctcgtagtatga |
| *DLG4 (PSD95)* | F-tcggtgacgacccatccat R-gcacgtccacttcatttacaaac |
| *SYN1* | F-cgcagtttggtcattgggc R-gtccccagtttcttatgcagtc |
| *SYN2* | F-catggcggagaatgaggactt R-tggttggggtagtatgtctgttc |
| *SYN3* | F-agcctttttagctccctctcc R-cactcggatctcaatctctcca |
| *SYNGAP1* | F-cgagtccagtcgcaacaaact R-gatggagctttttagccgtcg |
| *STX1A* | F-cgagaccgcttcatggatgag R-ttgaggacctctgtgtatttgtcaa |
| *RPL13A* | F-cctggaggagaagaggaaagaga R-ttgaggacctctgtgtatttgtcaa |
| *POLR2A* | F-gcccacgtccaatgacat R-gtgcggctgcttccataa |
| *RPL32* | F-caccagtcagaccgatatgtcaaaa R-tgttgtcaatgcctctgggttt |
| *GAPDH* | F-cgctctctgctcctcctgtt R-ccatggtgtctgagcgatgt |

**Supplementary Table 5.** List of primers using for Real-Time PCR of cortical neuronal cultures

| **Gene (PROTEIN)** | **Sequence (5’-3’)** |
| --- | --- |
| *Tardp* (TDP-43) | F-cctagcagtggcctagcggagat R-accgtcccatcgtcttctgatggt |
| *Nos1ap* (NOS1AP) | F-acgaaagcgaggagcgtgac R-cccaactcctgccgctgtaa |
| *Nos1* | F-tcctcagccgccaaaacctg R-gttggtgtggagacgcacga |
| *Grin1* (GluN1) | F-ggagcgtgagtccaaggcag R-ccgggcttccatcagcagag |
| *Grin2A* (GluN2A) | F-aactacaaggccgggagggat R-tggtggcaaagatgtacccgc |
| *Grin2B* (GluN2B) | F-cggagctggcatccgaataca R-gggtgtcgagggtttgagac |
| *Dlg1* (SAP97) | F-atgcttctgacgacgagtgg R-tgactgccctttatctcctct |
| *Dlg2* (PSD93) | F-acaccagtcattcccagcaca R-ggctccccttccagggagat |
| *Dlg4* (PSD95) | F-aagacacgccccctctggaa R-cctgcaactcatatcctggggc |
| *Cpe* | F-ccaatcgacctccctgtcgc R-acgctgtaccatgcaccacc |
| *Syn1* | F-ctcaaagccagcccctcc R-gcgaaagacttcctcaggct |
| *Syn2* | F-ggtgtttgctcagatggtg R-tctctcggtgattggggtag |
| *Syn3* | F-tcgatgacgcccatacagac R-ggtaacataagcagccaggttc |
| *Stx1A* | F-aactggaggagctcatgtcgg R-gctgcgtcttccggatcctc |
| *Llgl*2 (Scribble) | F-ccagtcataaacggcggcct R-ggcctgcgcttgatctggatt |
| *Syngap1-Pan* | F-caaccggaagctggaagagt R-catcagcctgccaatgatgc |
| *Rpl34* | F-ggtgctcagaggcactcaggatg R-gtgctttcccaaccttcttggtg |

**RNAscope assay**

SH-SY5Y cells were plated in 6-well plates containing coverslips coated with 0.01% poly-L-lysine solution (Sigma-Aldrich). After 24 hours, cells were silenced twice with TDP-43 and control (siLUC) using Lipofectamine 3000 reagent (Invitrogen). Non-transfected cells were also plated in a 6-well and further treated with RNAscope control probes.

After 48h from the second round of silencing (72 hours from seeding non-transfected cells), cells were fixated with 3.2% PFA and progressively dehydrated with EtOH at 50%-70%-100% concentration to be stored up to 6 months at -20°C.

The day of the experiment, coverslips were let air dried, fixed face up on a microscope slide and progressively rehydrated with EtOH at concentration of 100%-70%-50%. In order to achieve an optimal probe hybridization, slides were then treated with hydrogen peroxide for 10 minutes at room temperature and successively dark-incubated with protease III solution for 10 minutes at room temperature. Incubation with *B. subtilis* RNAscope Negative Control Probe-DapB (ACDbio), human RNAscope Positive Control Probe-Hs POLR2A (ACDbio) and human RNAscope Probe Hs-NOS1AP (ACDbio) was performed at 40°C for 2 hours in a HybEZ™ II Oven (ACDbio). RNAscope assay was finally carried out using the RNAscope 2.5 HD Detection Kit-RED (ACDbio), according to the manufacturer’s instructions. To counterstain the slides, 50% Gill’s Hematoxylin I (Bio-Optica) was used. Slides were then mounted using ProLong Gold antifade reagent with DAPI (Invitrogen). Images were acquired with Axioscope 5 microscope (Zeiss) equipped with Axiocam 208 color camera (Zeiss) and a 63X objective.

**Immunofluorescence analysis**

**﻿**SH-SY5Y cells were plated in 6-well plates containing coverslips coated with 0.01% (w/v) poly-L-lysine solution (Sigma-Aldrich). The day after, cells were silenced against NOS1AP and its relative control, as described in the previous section. After 72 hours, cells were washed three times with PBS, fixed in 3.2% paraformaldehyde (PFA) in 1X PBS for 1 hour at room temperature and permeabilized by using 0.3% Triton in 1X PBS for 5 minutes on ice. Cells were then blocked with 2% BSA/1X PBS for 20 minutes at room temperature and immunolabeled with 1:200 rabbit polyclonal anti-TDP-43 antibody (Proteintech 10782-AP) or 1:200 rabbit polyclonal anti-Phosho-TDP-43 (Ser409/410) (Proteintech 22309-1-AP) in 2% BSA/PBS overnight at 4°C. Next day, cells were washed three times with 1X PBS, incubated with 1:500 anti-rabbit Alexa-Fluor 488 (Invitrogen) for 1 hour at room temperature and mounted on microscope slides using ProLong Gold antifade reagent with DAPI (Invitrogen). Slides were analyzed using Axioscope 5 microscope (Zeiss) equipped with Axiocam 202 monocolor camera (Zeiss) and a 63X objective.

**Full-size and uncropped western blot images**

As described in the “Protein expression analysis” section of Materials and Methods, the images were acquired and analyzed using Alliance 9.7 Western Blot Imaging System (UVITEC, Cambridge).

**
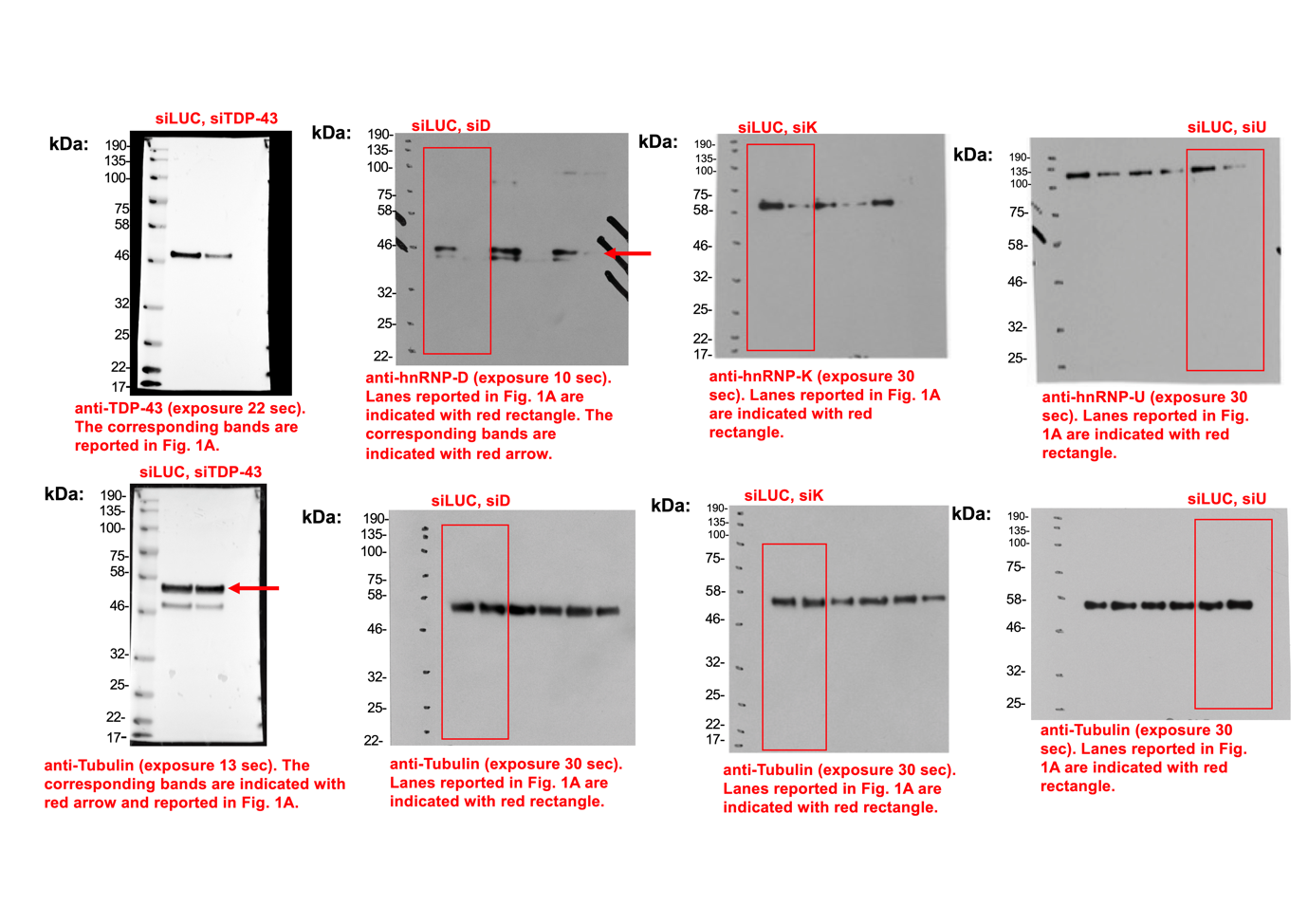
**

**Supplementary Fig. 10.** Uncropped gels related to Fig. 1A. Notably, Western Blotting images reported in Fig. 1A regarding hnRNP-D, hnRNP-K and hnRNP-U were developed in darkroom.


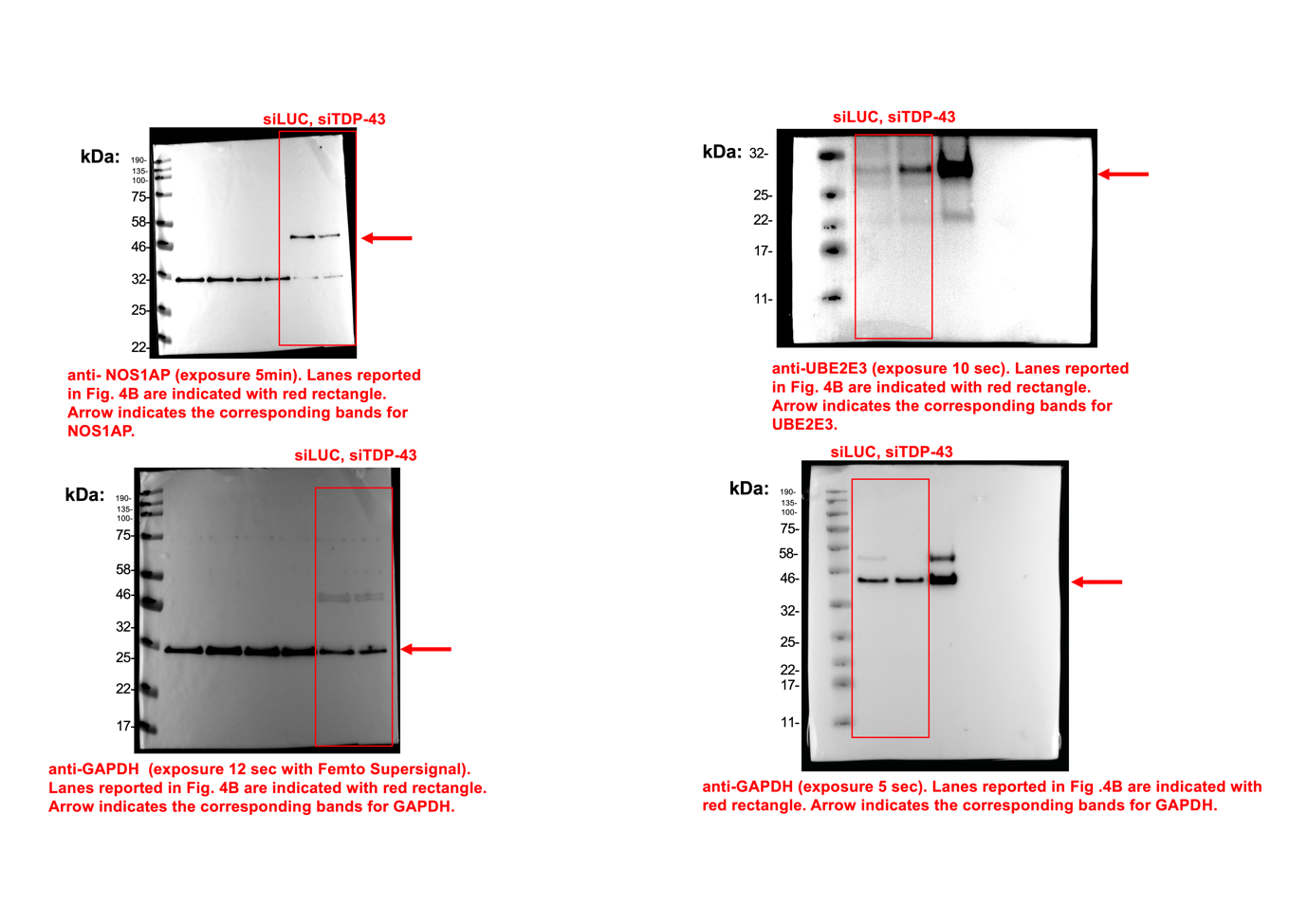
 **Supplementary Fig. 11.** Uncropped gels related to Fig. 4B.


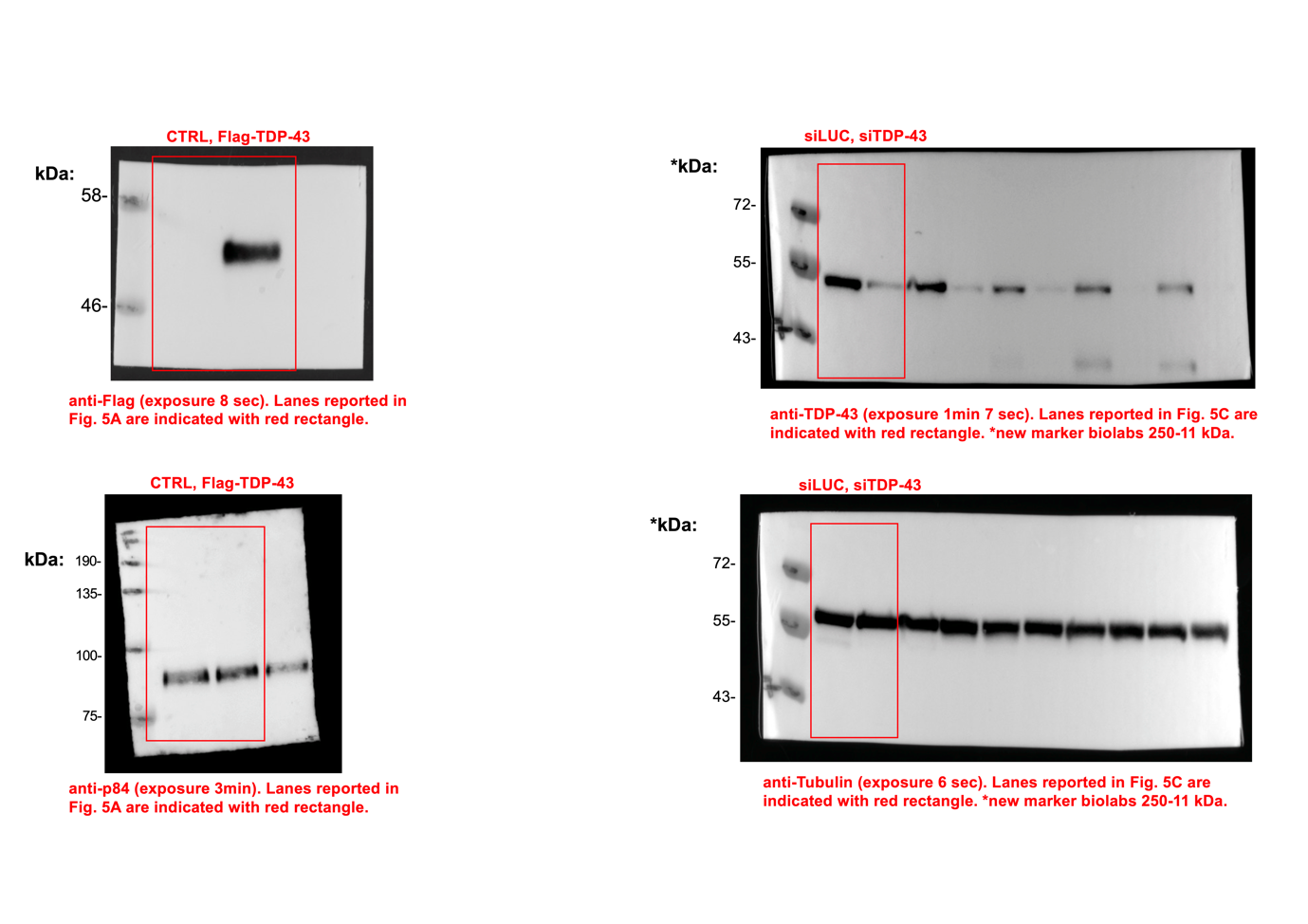
 **Supplementary Fig. 12.** Uncropped gels related to Fig. 5A (on the right) and Fig. 5B (on the left).

**
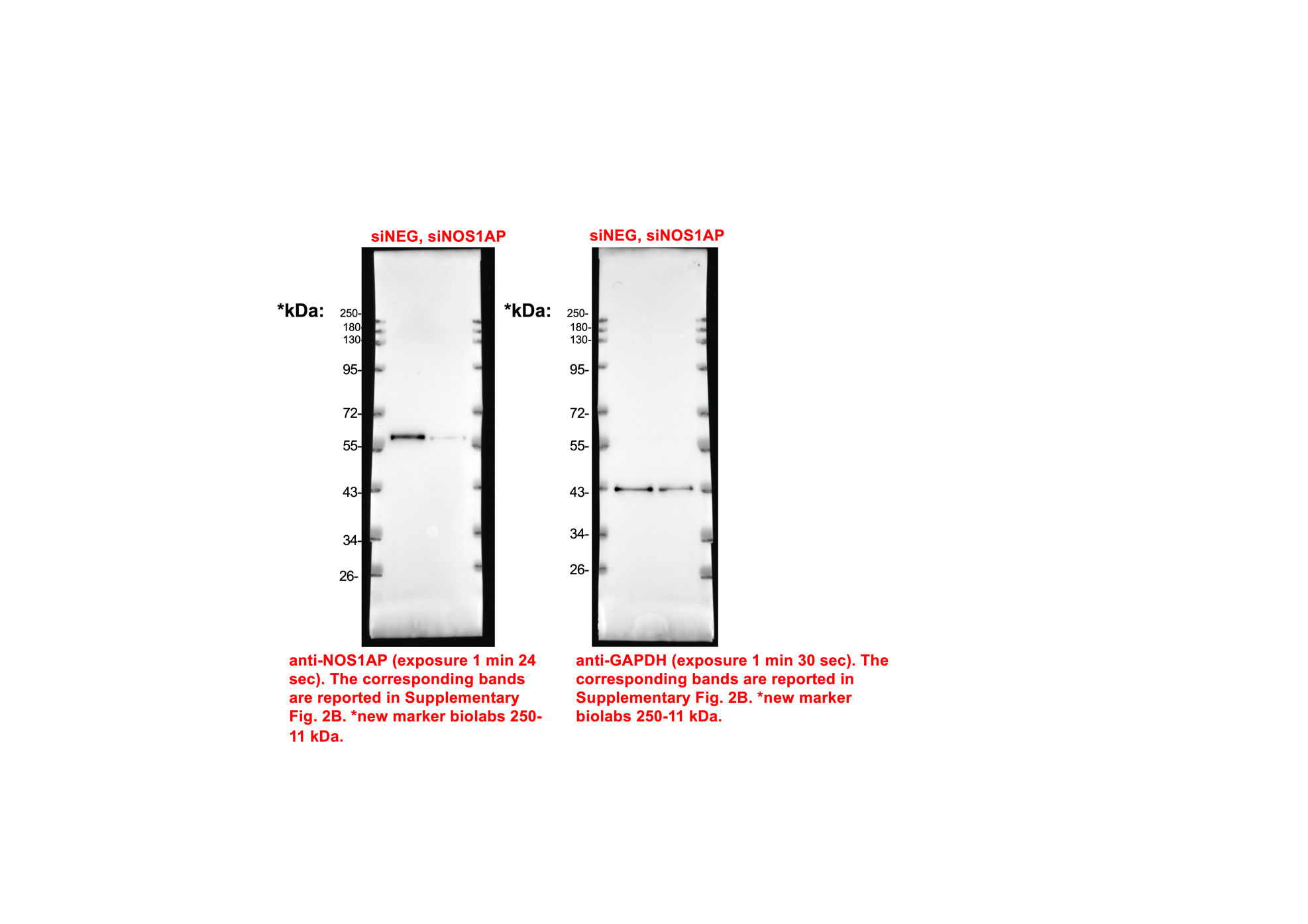
**

**Supplementary Fig. 13.** Uncropped gels related to Supplementary Fig. 2B.

**References**

1. Nutini M, Frazzini V, Marini C, Spalloni A, Sensi SL, Longone P. Zinc pre-treatment enhances NMDAR-mediated excitotoxicity in cultured cortical neurons from SOD1(G93A) mouse, a model of amyotrophic lateral sclerosis. *Neuropharmacology*. 2011;60(7-8):1200-1208. doi:10.1016/J.NEUROPHARM.2010.11.001
