# Supplementary material for: NOS1AP is a novel molecular target and critical factor in TDP-43 pathology
Source: Brain Commun. 2022 Sep 23;4(5):fcac242. doi: 10.1093/braincomms/fcac242 (PMC9576154; doi:10.1093/braincomms/fcac242)
Supplement: fcac242_Supplementary_Data [file fcac242_supplementary_data.zip › Supplementary_Table1,2.docx]

**Supplementary Table 1.** List of 214 commonly regulated genes among TDP-43, DAZAP1, hnRNP Q but not hnRNP R.

| **ENSEMBL ID** | **Gene name** | **Gene description** | **Fold Chnage TDP-43** | **Fold Change DAZAP1** | **Fold Change hnRNP Q** | |
| --- | --- | --- | --- | --- | --- | --- |
| ENSG00000003249 | DBNDD1 | DBNDD1, dysbindin (dystrobrevin binding protein 1) domain containing 1 | 0.64 | 0.69 | 0.58 | |
| ENSG00000003436 | TFPI | TFPI, tissue factor pathway inhibitor (lipoprotein-associated coagulation inhibitor) | 1.49 | 1.51 | 1.91 | |
| ENSG00000007402 | CACNA2D2 | CACNA2D2, calcium channel, voltage-dependent, alpha 2/delta subunit 2 | 0.68 | 0.65 | 0.59 | |
| ENSG00000015532 | XYLT2 | XYLT2, xylosyltransferase II | 0.70 | 0.50 | 0.58 | |
| ENSG00000023839 | ABCC2 | ABCC2, ATP-binding cassette, sub-family C (CFTR/MRP), member 2 | 3.30 | 5.26 | 2.89 | |
| ENSG00000031691 | CENPQ | CENPQ, centromere protein Q | 1.53 | 1.62 | 0.70 | |
| ENSG00000033100 | CHPF2 | CHPF2, chondroitin polymerizing factor 2 | 1.35 | 1.87 | 1.51 | |
| ENSG00000042832 | TG | TG, thyroglobulin | 0.35 | 0.37 | 0.39 | |
| ENSG00000049249 | TNFRSF9 | TNFRSF9, tumor necrosis factor receptor superfamily, member 9 | 35.97 | 10.11 | 9.87 | |
| ENSG00000050628 | PTGER3 | PTGER3, prostaglandin E receptor 3 (subtype EP3) | 2.28 | 2.00 | 2.93 | |
| ENSG00000063438 | AHRR | AHRR, aryl-hydrocarbon receptor repressor | 3.14 | 2.93 | 3.76 | |
| ENSG00000067113 | PPAP2A | PPAP2A, phosphatidic acid phosphatase type 2A | 1.41 | 1.41 | 1.52 | |
| ENSG00000069431 | ABCC9 | ABCC9, ATP-binding cassette, sub-family C (CFTR/MRP), member 9 | 0.61 | 0.25 | 0.63 | |
| ENSG00000073670 | ADAM11 | ADAM11, ADAM metallopeptidase domain 11 | 0.67 | 2.17 | 0.66 | |
| ENSG00000080709 | KCNN2 | KCNN2, potassium intermediate/small conductance calcium-activated channel, subfamily N, member 2 | 1.94 | 3.97 | 2.56 | |
| ENSG00000084710 | EFR3B | EFR3B, EFR3 homolog B (S. cerevisiae) | 0.61 | 0.52 | 0.46 | |
| ENSG00000085449 | WDFY1 | WDFY1, WD repeat and FYVE domain containing 1 | 1.68 | 1.58 | 1.55 | |
| ENSG00000087074 | PPP1R15A | PPP1R15A, protein phosphatase 1, regulatory subunit 15A | 1.72 | 2.73 | 1.93 | |
| ENSG00000088280 | ASAP3 | ASAP3, ArfGAP with SH3 domain, ankyrin repeat and PH domain 3 | 0.62 | 0.69 | 0.67 | |
| ENSG00000088727 | KIF9 | KIF9, kinesin family member 9 | 1.55 | 2.08 | 1.60 | |
| ENSG00000090263 | MRPS33 | MRPS33, mitochondrial ribosomal protein S33 | 1.36 | 1.31 | 1.45 | |
| ENSG00000090339 | ICAM1 | ICAM1, intercellular adhesion molecule 1 | 50.03 | 14.55 | 17.45 | |
| ENSG00000090612 | ZNF268 | ZNF268, zinc finger protein 268 | 1.69 | 2.11 | 1.44 | |
| ENSG00000092929 | UNC13D | UNC13D, unc-13 homolog D (C. elegans) | 0.54 | 0.55 | 0.49 | |
| ENSG00000100034 | PPM1F | PPM1F, protein phosphatase, Mg2+/Mn2+ dependent, 1F | 0.68 | 0.70 | 1.33 | |
| ENSG00000100078 | PLA2G3 | PLA2G3, phospholipase A2, group III | 0.35 | 0.26 | 0.35 | |
| ENSG00000100092 | SH3BP1 | SH3BP1, SH3-domain binding protein 1 | 0.58 | 0.64 | 0.60 | |
| ENSG00000100906 | NFKBIA | NFKBIA, nuclear factor of kappa light polypeptide gene enhancer in B-cells inhibitor, alpha | 1.58 | 1.47 | 1.54 | |
| ENSG00000101115 | SALL4 | SALL4, spalt-like transcription factor 4 | 0.61 | 0.57 | 1.48 | |
| ENSG00000101986 | ABCD1 | ABCD1, ATP-binding cassette, sub-family D (ALD), member 1 | 0.63 | 1.35 | 0.58 | |
| ENSG00000104967 | NOVA2 | NOVA2, neuro-oncological ventral antigen 2 | 0.38 | 0.67 | 0.65 | |
| ENSG00000105708 | ZNF14 | ZNF14, zinc finger protein 14 | 1.59 | 2.00 | 1.78 | |
| ENSG00000106070 | GRB10 | GRB10, growth factor receptor-bound protein 10 | 5.37 | 2.03 | 2.25 | |
| ENSG00000106366 | SERPINE1 | SERPINE1, serpin peptidase inhibitor, clade E (nexin, plasminogen activator inhibitor type 1), member 1 | 4.37 | 4.51 | 7.85 | |
| ENSG00000106617 | PRKAG2 | PRKAG2, protein kinase, AMP-activated, gamma 2 non-catalytic subunit | 1.42 | 1.46 | 0.66 | |
| ENSG00000106853 | PTGR1 | PTGR1, prostaglandin reductase 1 | 1.56 | 1.46 | 2.02 | |
| ENSG00000107165 | TYRP1 | TYRP1, tyrosinase-related protein 1 | 1.61 | 1.95 | 1.73 | |
| ENSG00000107201 | DDX58 | DDX58, DEAD (Asp-Glu-Ala-Asp) box polypeptide 58 | 1.91 | 5.15 | 2.32 | |
| ENSG00000108433 | GOSR2 | GOSR2, golgi SNAP receptor complex member 2 | 0.67 | 1.40 | 1.35 | |
| ENSG00000108733 | PEX12 | PEX12, peroxisomal biogenesis factor 12 | 1.57 | 1.44 | 1.51 | |
| ENSG00000108846 | ABCC3 | ABCC3, ATP-binding cassette, sub-family C (CFTR/MRP), member 3 | 3.51 | 3.12 | 2.32 | |
| ENSG00000112773 | FAM46A | FAM46A, family with sequence similarity 46, member A | 1.33 | 1.54 | 1.95 | |
| ENSG00000114670 | NEK11 | NEK11, NIMA-related kinase 11 | 3.34 | 8.74 | 2.86 | |
| ENSG00000114923 | SLC4A3 | SLC4A3, solute carrier family 4 (anion exchanger), member 3 | 1.65 | 1.47 | 0.56 | |
| ENSG00000115665 | SLC5A7 | SLC5A7, solute carrier family 5 (sodium/choline cotransporter), member 7 | 2.02 | 0.49 | 2.01 | |
| ENSG00000115756 | HPCAL1 | HPCAL1, hippocalcin-like 1 | 0.52 | 0.52 | 1.59 | |
| ENSG00000116574 | RHOU | RHOU, ras homolog family member U | 1.47 | 0.53 | 0.70 | |
| ENSG00000116981 | NT5C1A | NT5C1A, 5'-nucleotidase, cytosolic IA | 0.66 | 0.61 | 0.63 | |
| ENSG00000117586 | TNFSF4 | TNFSF4, tumor necrosis factor (ligand) superfamily, member 4 | 1.73 | 4.47 | 2.07 | |
| ENSG00000118976 | OTUD4P1 | OTUD4P1, OTUD4 pseudogene 1 | 1.66 | 1.50 | 1.83 | |
| ENSG00000121039 | RDH10 | RDH10, retinol dehydrogenase 10 (all-trans) | 0.67 | 1.51 | 1.39 | |
| ENSG00000122254 | HS3ST2 | HS3ST2, heparan sulfate (glucosamine) 3-O-sulfotransferase 2 | 0.66 | 0.43 | 0.67 | |
| ENSG00000122861 | PLAU | PLAU, plasminogen activator, urokinase | 8.66 | 6.50 | 2.70 | |
| ENSG00000123091 | RNF11 | RNF11, ring finger protein 11 | 0.55 | 0.45 | 0.69 | |
| ENSG00000125246 | CLYBL | CLYBL, citrate lyase beta like | 3.08 | 2.38 | 2.64 | |
| ENSG00000125520 | SLC2A4RG | SLC2A4RG, SLC2A4 regulator | 0.65 | 0.27 | 0.60 | |
| ENSG00000126947 | ARMCX1 | ARMCX1, armadillo repeat containing, X-linked 1 | 1.38 | 0.61 | 1.35 | |
| ENSG00000127561 | SYNGR3 | SYNGR3, synaptogyrin 3 | 1.84 | 2.29 | 0.60 | |
| ENSG00000128016 | ZFP36 | ZFP36, ZFP36 ring finger protein | 1.82 | 3.70 | 1.52 | |
| ENSG00000128482 | RNF112 | RNF112, ring finger protein 112 | 0.67 | 0.52 | 0.59 | |
| ENSG00000128923 | FAM63B | FAM63B, family with sequence similarity 63, member B | 1.65 | 1.42 | 0.36 | |
| ENSG00000130176 | CNN1 | CNN1, calponin 1, basic, smooth muscle | 0.46 | 0.63 | 2.19 | |
| ENSG00000130600 | H19 | H19, H19, imprinted maternally expressed transcript (non-protein coding) | 0.43 | 0.18 | 0.57 | |
| ENSG00000130762 | ARHGEF16 | ARHGEF16, Rho guanine nucleotide exchange factor (GEF) 16 | 2.55 | 1.95 | 0.48 | |
| ENSG00000130772 | MED18 | MED18, mediator complex subunit 18 | 1.30 | 1.53 | 1.55 | |
| ENSG00000132141 | CCT6B | CCT6B, chaperonin containing TCP1, subunit 6B (zeta 2) | 0.45 | 4.47 | 0.55 | |
| ENSG00000132824 | SERINC3 | SERINC3, serine incorporator 3 | 1.50 | 1.39 | 1.39 | |
| ENSG00000133997 | MED6 | MED6, mediator complex subunit 6 | 0.69 | 1.64 | 1.34 | |
| ENSG00000134070 | IRAK2 | IRAK2, interleukin-1 receptor-associated kinase 2 | 6.48 | 4.66 | 3.34 | |
| ENSG00000134326 | CMPK2 | CMPK2, cytidine monophosphate (UMP-CMP) kinase 2, mitochondrial | 1.66 | 2.05 | 1.85 | |
| ENSG00000135048 | TMEM2 | TMEM2, transmembrane protein 2 | 2.21 | 1.99 | 1.91 | |
| ENSG00000135097 | MSI1 | MSI1, musashi RNA-binding protein 1 | 0.58 | 0.50 | 0.47 | |
| ENSG00000135318 | NT5E | NT5E, 5'-nucleotidase, ecto (CD73) | 1.85 | 1.85 | 3.84 | |
| ENSG00000135776 | ABCB10 | ABCB10, ATP-binding cassette, sub-family B (MDR/TAP), member 10 | 1.58 | 0.44 | 0.64 | |
| ENSG00000135926 | TMBIM1 | TMBIM1, transmembrane BAX inhibitor motif containing 1 | 1.95 | 0.40 | 2.04 | |
| ENSG00000136052 | SLC41A2 | SLC41A2, solute carrier family 41 (magnesium transporter), member 2 | 2.84 | 2.88 | 0.51 | |
| ENSG00000136167 | LCP1 | LCP1, lymphocyte cytosolic protein 1 (L-plastin) | 1.40 | 3.68 | 2.74 | |
| ENSG00000136895 | GARNL3 | GARNL3, GTPase activating Rap/RanGAP domain-like 3 | 1.32 | 0.70 | 1.37 | |
| ENSG00000137331 | IER3 | IER3, immediate early response 3 | 3.07 | 3.03 | 1.56 | |
| ENSG00000138078 | PREPL | PREPL, prolyl endopeptidase-like | 1.50 | 1.52 | 1.42 | |
| ENSG00000138760 | SCARB2 | SCARB2, scavenger receptor class B, member 2 | 1.40 | 1.51 | 0.63 | |
| ENSG00000139117 | CPNE8 | CPNE8, copine VIII | 1.34 | 1.48 | 1.50 | |
| ENSG00000139200 | PIANP | PIANP, PILR alpha associated neural protein | 0.70 | 0.67 | 0.64 | |
| ENSG00000140511 | HAPLN3 | HAPLN3, hyaluronan and proteoglycan link protein 3 | 3.31 | 5.23 | 0.31 | |
| ENSG00000141750 | STAC2 | STAC2, SH3 and cysteine rich domain 2 | 0.68 | 0.31 | 0.65 | |
| ENSG00000141753 | IGFBP4 | IGFBP4, insulin-like growth factor binding protein 4 | 0.53 | 0.61 | 1.41 | |
| ENSG00000142102 | ATHL1 | ATHL1, ATH1, acid trehalase-like 1 (yeast) | 1.96 | 0.64 | 0.60 | |
| ENSG00000142197 | DOPEY2 | DOPEY2, dopey family member 2 | 0.51 | 0.52 | 0.52 | |
| ENSG00000142303 | ADAMTS10 | ADAMTS10, ADAM metallopeptidase with thrombospondin type 1 motif, 10 | 2.01 | 4.11 | 0.49 | |
| ENSG00000143469 | SYT14 | SYT14, synaptotagmin XIV | 1.43 | 1.38 | 0.70 | |
| ENSG00000144677 | CTDSPL | CTDSPL, CTD (carboxy-terminal domain, RNA polymerase II, polypeptide A) small phosphatase-like | 0.59 | 0.69 | 1.54 | |
| ENSG00000146094 | DOK3 | DOK3, docking protein 3 | 0.65 | 0.57 | 0.52 | |
| ENSG00000146592 | CREB5 | CREB5, cAMP responsive element binding protein 5 | 2.80 | 2.27 | 1.41 | |
| ENSG00000146966 | DENND2A | DENND2A, DENN/MADD domain containing 2A | 1.46 | 1.46 | 0.39 | |
| ENSG00000148180 | GSN | GSN, gelsolin | 2.66 | 1.93 | 2.09 | |
| ENSG00000148700 | ADD3 | ADD3, adducin 3 (gamma) | 0.64 | 0.69 | 0.51 | |
| ENSG00000149243 | KLHL35 | KLHL35, kelch-like family member 35 | 1.72 | 0.34 | 1.62 | |
| ENSG00000149260 | CAPN5 | CAPN5, calpain 5 | 0.68 | 0.57 | 0.66 | |
| ENSG00000149295 | DRD2 | DRD2, dopamine receptor D2 | 0.65 | 0.41 | 1.35 | |
| ENSG00000152518 | ZFP36L2 | ZFP36L2, ZFP36 ring finger protein-like 2 | 1.34 | 0.63 | 1.63 | |
| ENSG00000153214 | TMEM87B | TMEM87B, transmembrane protein 87B | 1.49 | 1.43 | 2.23 | |
| ENSG00000153363 | LINC00467 | LINC00467, long intergenic non-protein coding RNA 467 | 1.57 | 3.67 | 1.81 | |
| ENSG00000154997 | Sep-14 | SEPT14, septin 14 | 0.41 | 2.33 | 2.19 | |
| ENSG00000155729 | KCTD18 | KCTD18, potassium channel tetramerization domain containing 18 | 1.32 | 1.52 | 0.62 | |
| ENSG00000156966 | B3GNT7 | B3GNT7, UDP-GlcNAc:betaGal beta-1,3-N-acetylglucosaminyltransferase 7 | 2.08 | 0.32 | 2.13 | |
| ENSG00000157680 | DGKI | DGKI, diacylglycerol kinase, iota | 1.58 | 1.99 | 1.84 | |
| ENSG00000157703 | SVOPL | SVOPL, SVOP-like | 0.30 | 0.32 | 0.19 | |
| ENSG00000158373 | HIST1H2BD | HIST1H2BD, histone cluster 1, H2bd | 1.31 | 4.73 | 1.50 | |
| ENSG00000158470 | B4GALT5 | B4GALT5, UDP-Gal:betaGlcNAc beta 1,4- galactosyltransferase, polypeptide 5 | 1.33 | 1.33 | 1.87 | |
| ENSG00000159450 | TCHH | TCHH, trichohyalin | 4.24 | 6.57 | 5.91 | |
| ENSG00000159873 | CCDC117 | CCDC117, coiled-coil domain containing 117 | 0.62 | 0.59 | 1.33 | |
| ENSG00000161638 | ITGA5 | ITGA5, integrin, alpha 5 (fibronectin receptor, alpha polypeptide) | 2.76 | 3.80 | 2.60 | |
| ENSG00000162188 | GNG3 | GNG3, guanine nucleotide binding protein (G protein), gamma 3 | 0.40 | 0.44 | 0.63 | |
| ENSG00000162396 | PARS2 | PARS2, prolyl-tRNA synthetase 2, mitochondrial (putative) | 1.40 | 2.41 | 1.60 | |
| ENSG00000162616 | DNAJB4 | DNAJB4, DnaJ (Hsp40) homolog, subfamily B, member 4 | 1.73 | 2.60 | 1.54 | |
| ENSG00000162959 | MEMO1 | MEMO1, mediator of cell motility 1 | 0.70 | 0.61 | 0.51 | |
| ENSG00000163131 | CTSS | CTSS, cathepsin S | 3.94 | 6.03 | 4.37 | |
| ENSG00000163536 | SERPINI1 | SERPINI1, serpin peptidase inhibitor, clade I (neuroserpin), member 1 | 0.51 | 4.26 | 1.69 | |
| ENSG00000163820 | FYCO1 | FYCO1, FYVE and coiled-coil domain containing 1 | 1.78 | 0.38 | 1.44 | |
| ENSG00000164125 | FAM198B | FAM198B, family with sequence similarity 198, member B | 0.61 | 0.35 | 1.51 | |
| ENSG00000164305 | CASP3 | CASP3, caspase 3, apoptosis-related cysteine peptidase | 1.65 | 1.30 | 1.68 | |
| ENSG00000164742 | ADCY1 | ADCY1, adenylate cyclase 1 (brain) | 0.67 | 0.56 | 0.62 | |
| ENSG00000165238 | WNK2 | WNK2, WNK lysine deficient protein kinase 2 | 0.67 | 0.67 | 0.67 | |
| ENSG00000165424 | ZCCHC24 | ZCCHC24, zinc finger, CCHC domain containing 24 | 0.47 | 0.38 | 1.80 | |
| ENSG00000166192 | SENP8 | SENP8, SUMO/sentrin specific peptidase family member 8 | 1.64 | 1.50 | 1.61 | |
| ENSG00000166246 | C16orf71 | C16orf71, chromosome 16 open reading frame 71 | 2.21 | 3.97 | 2.48 | |
| ENSG00000166401 | SERPINB8 | SERPINB8, serpin peptidase inhibitor, clade B (ovalbumin), member 8 | 2.38 | 5.38 | 2.41 | |
| ENSG00000166432 | ZMAT1 | ZMAT1, zinc finger, matrin-type 1 | 1.83 | 6.55 | 0.40 | |
| ENSG00000167244 | IGF2 | IGF2, insulin-like growth factor 2 (somatomedin A) | 0.42 | 0.16 | 0.44 | |
| ENSG00000167524 | SGK494 | SGK494, uncharacterized serine/threonine-protein kinase SgK494 | 0.61 | 0.53 | 0.41 | |
| ENSG00000167740 | CYB5D2 | CYB5D2, cytochrome b5 domain containing 2 | 1.51 | 2.81 | 1.68 | |
| ENSG00000167889 | MGAT5B | MGAT5B, mannosyl (alpha-1,6-)-glycoprotein beta-1,6-N-acetyl-glucosaminyltransferase, isozyme B | 0.50 | 0.48 | 1.39 | |
| ENSG00000168026 | TTC21A | TTC21A, tetratricopeptide repeat domain 21A | 2.31 | 7.26 | 1.82 | |
| ENSG00000168398 | BDKRB2 | BDKRB2, bradykinin receptor B2 | 1.92 | 2.04 | 2.77 | |
| ENSG00000168461 | RAB31 | RAB31, RAB31, member RAS oncogene family | 1.96 | 2.53 | 1.47 | |
| ENSG00000168542 | COL3A1 | COL3A1, collagen, type III, alpha 1 | 2.41 | 1.96 | 3.06 | |
| ENSG00000169981 | ZNF35 | ZNF35, zinc finger protein 35 | 1.34 | 1.77 | 1.53 | |
| ENSG00000170035 | UBE2E3 | UBE2E3, ubiquitin-conjugating enzyme E2E 3 | 1.45 | 1.43 | 1.57 | |
| ENSG00000171295 | ZNF440 | ZNF440, zinc finger protein 440 | 1.31 | 1.45 | 1.51 | |
| ENSG00000171303 | KCNK3 | KCNK3, potassium channel, subfamily K, member 3 | 0.62 | 0.54 | 1.42 | |
| ENSG00000171310 | CHST11 | CHST11, carbohydrate (chondroitin 4) sulfotransferase 11 | 0.70 | 0.47 | 0.47 | |
| ENSG00000171365 | CLCN5 | CLCN5, chloride channel, voltage-sensitive 5 | 1.58 | 1.54 | 1.44 | |
| ENSG00000171451 | DSEL | DSEL, dermatan sulfate epimerase-like | 1.63 | 0.47 | 0.49 | |
| ENSG00000171992 | SYNPO | SYNPO, synaptopodin | 1.32 | 0.64 | 0.59 | |
| ENSG00000172752 | COL6A5 | COL6A5, collagen, type VI, alpha 5 | 4.03 | 2.70 | 2.85 | |
| ENSG00000172794 | RAB37 | RAB37, RAB37, member RAS oncogene family | 0.67 | 0.69 | 1.42 | |
| ENSG00000173517 | PEAK1 | PEAK1, pseudopodium-enriched atypical kinase 1 | 0.53 | 2.34 | 0.59 | |
| ENSG00000173621 | LRFN4 | LRFN4, leucine rich repeat and fibronectin type III domain containing 4 | 0.66 | 0.64 | 0.61 | |
| ENSG00000174672 | BRSK2 | BRSK2, BR serine/threonine kinase 2 | 0.65 | 0.61 | 0.67 | |
| ENSG00000175093 | SPSB4 | SPSB4, splA/ryanodine receptor domain and SOCS box containing 4 | 0.61 | 0.55 | 0.55 | |
| ENSG00000175229 | GAL3ST3 | GAL3ST3, galactose-3-O-sulfotransferase 3 | 0.62 | 0.49 | 0.62 | |
| ENSG00000175592 | FOSL1 | FOSL1, FOS-like antigen 1 | 2.76 | 4.02 | 4.30 | |
| ENSG00000175785 | PRIMA1 | PRIMA1, proline rich membrane anchor 1 | 0.47 | 0.33 | 0.59 | |
| ENSG00000176749 | CDK5R1 | CDK5R1, cyclin-dependent kinase 5, regulatory subunit 1 (p35) | 0.65 | 0.38 | 0.61 | |
| ENSG00000177239 | MAN1B1 | MAN1B1, mannosidase, alpha, class 1B, member 1 | 1.33 | 1.56 | 1.35 | |
| ENSG00000177272 | KCNA3 | KCNA3, potassium voltage-gated channel, shaker-related subfamily, member 3 | 1.64 | 2.28 | 2.87 | |
| ENSG00000177830 | CHID1 | CHID1, chitinase domain containing 1 | 0.59 | 1.48 | 1.33 | |
| ENSG00000179242 | CDH4 | CDH4, cadherin 4, type 1, R-cadherin (retinal) | 0.57 | 0.47 | 1.68 | |
| ENSG00000179921 | GPBAR1 | GPBAR1, G protein-coupled bile acid receptor 1 | 0.49 | 0.17 | 1.81 | |
| ENSG00000181201 | HIST3H2BA | HIST3H2BA, histone cluster 3, H2ba, pseudogene | 0.06 | 3.61 | 0.14 | |
| ENSG00000181523 | SGSH | SGSH, N-sulfoglucosamine sulfohydrolase | 1.34 | 1.31 | 1.66 | |
| ENSG00000183044 | ABAT | ABAT, 4-aminobutyrate aminotransferase | 0.60 | 2.07 | 0.38 | |
| ENSG00000183696 | UPP1 | UPP1, uridine phosphorylase 1 | 2.51 | 2.13 | 1.58 | |
| ENSG00000185658 | BRWD1 | BRWD1, bromodomain and WD repeat domain containing 1 | 1.31 | 1.79 | 1.31 | |
| ENSG00000185900 | POMK | POMK, protein-O-mannose kinase | 0.58 | 0.51 | 0.40 | |
| ENSG00000185947 | ZNF267 | ZNF267, zinc finger protein 267 | 1.44 | 1.57 | 1.65 | |
| ENSG00000186866 | POFUT2 | POFUT2, protein O-fucosyltransferase 2 | 1.51 | 1.65 | 1.48 | |
| ENSG00000187164 | KIAA1598 | KIAA1598, KIAA1598 | 7.56 | 7.62 | 2.33 | |
| ENSG00000188042 | ARL4C | ARL4C, ADP-ribosylation factor-like 4C | 1.37 | 0.59 | 1.69 | |
| ENSG00000196437 | ZNF569 | ZNF569, zinc finger protein 569 | 1.42 | 1.65 | 0.68 | |
| ENSG00000196664 | TLR7 | TLR7, toll-like receptor 7 | 7.04 | 3.99 | 4.64 | |
| ENSG00000197122 | SRC | SRC, SRC proto-oncogene, non-receptor tyrosine kinase | 0.65 | 0.65 | 0.61 | |
| ENSG00000197586 | ENTPD6 | ENTPD6, ectonucleoside triphosphate diphosphohydrolase 6 (putative) | 0.66 | 0.64 | 1.31 | |
| ENSG00000198046 | ZNF667 | ZNF667, zinc finger protein 667 | 1.35 | 1.64 | 1.44 | |
| ENSG00000198168 | SVIP | SVIP, small VCP/p97-interacting protein | 0.65 | 0.47 | 0.63 | |
| ENSG00000198570 | RD3 | RD3, retinal degeneration 3 | 0.55 | 0.23 | 0.49 | |
| ENSG00000198929 | NOS1AP | NOS1AP, nitric oxide synthase 1 (neuronal) adaptor protein | 0.59 | 0.62 | 0.68 | |
| ENSG00000204248 | COL11A2 | COL11A2, collagen, type XI, alpha 2 | 5.76 | | 12.21 | 3.04 |
| ENSG00000205625 | RP11-15G8.1 | RP11-15G8.1 | 0.24 | | 0.65 | 0.68 |
| ENSG00000206838 | SNORA5A | SNORA5A, small nucleolar RNA, H/ACA box 5A | 0.40 | | 0.36 | 0.49 |
| ENSG00000210107 | MT-TQ | MT-TQ, mitochondrially encoded tRNA glutamine | 1.56 | | 1.83 | 1.59 |
| ENSG00000223427 | AC016716.1 | AC016716.1 | 1.91 | | 0.50 | 1.56 |
| ENSG00000224165 | DNAJC27-AS1 | DNAJC27-AS1, DNAJC27 antisense RNA 1 | 2.89 | | 21.32 | 2.97 |
| ENSG00000228876 | AC010745.2 | AC010745.2 | 1.75 | | 0.37 | 1.62 |
| ENSG00000230138 | RP11-117D22.2 | RP11-117D22.2 | 0.38 | | 0.30 | 0.23 |
| ENSG00000232810 | TNF | TNF, tumor necrosis factor | 20.54 | | 11.18 | 24.52 |
| ENSG00000238105 | GOLGA2P5 | GOLGA2P5, golgin A2 pseudogene 5 | 1.49 | | 1.75 | 0.63 |
| ENSG00000239887 | C1orf226 | C1orf226, chromosome 1 open reading frame 226 | 0.65 | | 0.54 | 0.66 |
| ENSG00000240065 | PSMB9 | PSMB9, proteasome (prosome, macropain) subunit, beta type, 9 | 4.43 | | 8.04 | 0.12 |
| ENSG00000247157 | LINC01252 | LINC01252, long intergenic non-protein coding RNA 1252 | 2.83 | | 3.55 | 4.40 |
| ENSG00000248323 | LUCAT1 | LUCAT1, lung cancer associated transcript 1 (non-protein coding) | 2.18 | | 2.47 | 0.41 |
| ENSG00000248383 | PCDHAC1 | PCDHAC1, protocadherin alpha subfamily C, 1 | 1.42 | | 2.13 | 2.16 |
| ENSG00000248905 | FMN1 | FMN1, formin 1 | 2.29 | | 1.58 | 1.74 |
| ENSG00000250091 | DNAH10OS | DNAH10OS, dynein, axonemal, heavy chain 10 opposite strand | 1.44 | | 1.31 | 0.31 |
| ENSG00000253869 | PIGFP1 | PIGFP1, phosphatidylinositol glycan anchor biosynthesis, class F, pseudogene 1 | 1.68 | | 1.71 | 1.59 |
| ENSG00000254473 | RP11-522I20.3 | RP11-522I20.3 | 1.98 | | 8.84 | 2.94 |
| ENSG00000256304 | CCDC150P1 | CCDC150P1, coiled-coil domain containing 150 pseudogene 1 | 1.63 | | 0.45 | 0.60 |
| ENSG00000258472 | RP11-192H23.4 | RP11-192H23.4, Uncharacterized protein | 0.37 | | 0.21 | 0.17 |
| ENSG00000259291 | RP11-617F23.1 | RP11-617F23.1 | 0.68 | | 0.38 | 0.47 |
| ENSG00000259381 | RP11-192M23.1 | RP11-192M23.1 | 2.17 | | 2.00 | 2.03 |
| ENSG00000259834 | KCNA3 | KCNA3, potassium voltage-gated channel, shaker-related subfamily, member 3 | 2.19 | | 2.26 | 2.93 |
| ENSG00000261762 | RP11-650L12.2 | RP11-650L12.2 | 0.67 | | 0.35 | 0.55 |
| ENSG00000265971 | RP11-269G24.6 | RP11-269G24.6 | 0.48 | | 0.42 | 0.39 |
| ENSG00000266921 | RP11-15A1.7 | RP11-15A1.7 | 3.74 | | 13.41 | 3.91 |
| ENSG00000267682 | CTD-3220F14.2 | CTD-3220F14.2 | 2.49 | | 3.15 | 3.04 |
| ENSG00000267980 | AC007292.6 | AC007292.6 | 0.56 | | 0.58 | 0.62 |
| ENSG00000270015 | RP11-540B6.6 | RP11-540B6.6 | 0.60 | | 1.35 | 1.39 |
| ENSG00000271155 | RP11-435O5.5 | RP11-435O5.5 | 0.56 | | 2.70 | 0.56 |
| ENSG00000272947 | RP11-71H17.9 | RP11-71H17.9 | 1.47 | | 1.51 | 0.70 |
| ENSG00000273820 | USP27X | USP27X, ubiquitin specific peptidase 27, X-linked | 1.44 | | 1.58 | 1.37 |
| ENSG00000276603 | RP11-425M5.7 | RP11-425M5.7 | 2.32 | | 8.99 | 2.12 |
| ENSG00000278619 | MRM1 | MRM1, mitochondrial rRNA methyltransferase 1 homolog (S. cerevisiae) | 0.68 | | 0.50 | 1.34 |
| ENSG00000279192 | PWAR5 | PWAR5, Prader Willi/Angelman region RNA 5 | 1.57 | | 1.88 | 1.64 |
| ENSG00000280287 | RP13-554M15.7 | RP13-554M15.7 | 1.66 | | 10.73 | 2.28 |

**Supplementary Table 2.** List of 52 commonly regulated genes among TDP-43, hnRNP D, hnRNP K and hnRNP U.

| **ENSEMBL ID** | **Gene name** | **Gene description** | **Fold Chnage TDP-43** | **Fold Change hnRNP D** | **Fold Chnage hnRNP K** | **Fold Change hnRNP U** |
| --- | --- | --- | --- | --- | --- | --- |
| ENSG00000015592 | STMN4 | stathmin-like 4 | 2.65 | 0.10 | 0.31 | 0.35 |
| ENSG00000023445 | BIRC3 | baculoviral IAP repeat containing 3 | 4.02 | 1.64 | 3.45 | 1.30 |
| ENSG00000033100 | CHPF2 | chondroitin polymerizing factor 2 | 1.35 | 1.42 | 1.51 | 0.58 |
| ENSG00000072682 | P4HA2 | prolyl 4-hydroxylase alpha polypeptide II | 1.45 | 0.67 | 1.36 | 1.46 |
| ENSG00000087303 | NID2 | nidogen 2 (osteonidogen) | 0.51 | 1.77 | 2.59 | 1.56 |
| ENSG00000095370 | SH2D3C | SH2 domain containing 3C | 1.43 | 0.51 | 0.62 | 0.54 |
| ENSG00000096093 | EFHC1 | EF-hand domain (C-terminal) containing 1 | 1.58 | 1.63 | 1.72 | 1.38 |
| ENSG00000103642 | LACTB | lactamase beta | 1.45 | 0.68 | 0.64 | 1.34 |
| ENSG00000105251 | SHD | Src homology 2 domain containing transforming protein D | 0.54 | 1.55 | 2.01 | 0.63 |
| ENSG00000108799 | EZH1 | enhancer of zeste homolog 1 (Drosophila) | 1.41 | 0.67 | 0.62 | 1.34 |
| ENSG00000117154 | IGSF21 | immunoglobin superfamily member 21 | 0.39 | 0.56 | 1.44 | 0.46 |
| ENSG00000121440 | PDZRN3 | PDZ domain containing ring finger 3 | 0.50 | 1.46 | 1.40 | 0.68 |
| ENSG00000123201 | GUCY1B2 | guanylate cyclase 1 soluble beta 2 (pseudogene) | 1.81 | 1.36 | 1.48 | 0.63 |
| ENSG00000123240 | OPTN | optineurin | 2.21 | 0.66 | 1.49 | 1.46 |
| ENSG00000125869 | LAMP5 | lysosomal-associated membrane protein family member 5 | 0.54 | 1.64 | 2.76 | 0.58 |
| ENSG00000127080 | IPPK | inositol 13456-pentakisphosphate 2-kinase | 1.63 | 1.48 | 1.38 | 1.38 |
| ENSG00000128482 | RNF112 | ring finger protein 112 | 0.67 | 0.58 | 1.90 | 0.62 |
| ENSG00000130487 | KLHDC7B | kelch domain containing 7B | 16.64 | 0.35 | 0.40 | 0.46 |
| ENSG00000131409 | LRRC4B | leucine rich repeat containing 4B | 2.09 | 0.60 | 1.56 | 0.70 |
| ENSG00000131634 | TMEM204 | transmembrane protein 204 | 0.61 | 1.49 | 1.89 | 0.70 |
| ENSG00000134070 | IRAK2 | interleukin-1 receptor-associated kinase 2 | 6.48 | 0.54 | 2.65 | 1.44 |
| ENSG00000136720 | HS6ST1 | heparan sulfate 6-O-sulfotransferase 1 | 0.64 | 1.71 | 1.90 | 0.69 |
| ENSG00000137507 | LRRC32 | leucine rich repeat containing 32 | 0.33 | 0.50 | 1.97 | 0.55 |
| ENSG00000149269 | PAK1 | p21 protein (Cdc42/Rac)-activated kinase 1 | 2.63 | 0.66 | 2.24 | 1.30 |
| ENSG00000149557 | FEZ1 | fasciculation and elongation protein zeta 1 (zygin I) | 3.83 | 0.39 | 2.59 | 0.42 |
| ENSG00000150672 | DLG2 | discs large homolog 2 (Drosophila) | 0.68 | 0.64 | 0.42 | 0.50 |
| ENSG00000155792 | DEPTOR | DEP domain containing MTOR-interacting protein | 1.50 | 0.68 | 0.56 | 1.45 |
| ENSG00000161082 | CELF5 | CUGBP Elav-like family member 5 | 4.59 | 0.46 | 1.77 | 0.49 |
| ENSG00000162706 | CADM3 | cell adhesion molecule 3 | 2.64 | 0.50 | 2.14 | 0.66 |
| ENSG00000163273 | NPPC | natriuretic peptide C | 0.38 | 0.65 | 1.76 | 0.58 |
| ENSG00000163328 | GPR155 | G protein-coupled receptor 155 | 0.65 | 0.64 | 0.36 | 0.69 |
| ENSG00000166900 | STX3 | syntaxin 3 | 5.49 | 0.63 | 3.21 | 1.95 |
| ENSG00000167103 | PIP5KL1 | phosphatidylinositol-4-phosphate 5-kinase-like 1 | 2.74 | 0.39 | 0.42 | 0.62 |
| ENSG00000167244 | IGF2 | insulin-like growth factor 2 (somatomedin A) | 0.42 | 2.21 | 7.45 | 0.45 |
| ENSG00000167995 | BEST1 | bestrophin_1 | 2.85 | 0.39 | 0.57 | 0.42 |
| ENSG00000168394 | TAP1 | transporter 1 ATP-binding cassette sub-family B (MDR/TAP) | 1.66 | 1.36 | 1.68 | 1.31 |
| ENSG00000170035 | UBE2E3 | ubiquitin-conjugating enzyme E2 E3 | 1.45 | 1.93 | 1.53 | 1.69 |
| ENSG00000177791 | MYOZ1 | myozenin 1 | 6.83 | 0.57 | 1.98 | 1.59 |
| ENSG00000180287 | PLD5 | phospholipase D family member 5 | 0.57 | 0.37 | 2.03 | 0.49 |
| ENSG00000181409 | AATK | apoptosis-associated tyrosine kinase | 0.66 | 0.56 | 0.65 | 0.65 |
| ENSG00000181444 | ZNF467 | zinc finger protein 467 | 0.36 | 0.60 | 2.18 | 1.45 |
| ENSG00000196646 | ZNF136 | zinc finger protein 136 | 1.46 | 0.67 | 1.35 | 1.32 |
| ENSG00000198929 | NOS1AP | nitric oxide synthase 1 (neuronal) adaptor protein | 0.59 | 0.49 | 0.45 | 0.58 |
| ENSG00000205336 | GPR56 | G protein-coupled receptor 56 | 4.00 | 0.55 | 2.42 | 0.57 |
| ENSG00000213626 | LBH | limb bud and heart development | 0.60 | 0.59 | 0.54 | 0.69 |
| ENSG00000226041 | AC010745.1 | - | 1.95 | 2.16 | 2.14 | 1.40 |
| ENSG00000226777 | KIAA0125 | KIAA0125 | 0.49 | 2.33 | 4.72 | 0.57 |
| ENSG00000230487 | PSMG3-AS1 | PSMG3 antisense RNA 1 (head to head) | 1.53 | 2.07 | 1.81 | 0.64 |
| ENSG00000239887 | C1orf226 | chromosome 1 open reading frame 226 | 0.65 | 0.56 | 0.51 | 0.56 |
| ENSG00000250033 | SLC7A11-AS1 | SLC7A11 antisense RNA 1 | 8.10 | 0.55 | 0.47 | 1.41 |
| ENSG00000259518 | RP11-597K23.2 | Uncharacterized protein | 9.49 | 0.29 | 0.48 | 0.67 |
| ENSG00000268460 | AC006262.6 | - | 2.77 | 0.63 | 4.07 | 0.69 |
