# Supplementary material for: NOS1AP is a novel molecular target and critical factor in TDP-43 pathology
Source: Brain Commun. 2022 Sep 23;4(5):fcac242. doi: 10.1093/braincomms/fcac242 (PMC9576154; doi:10.1093/braincomms/fcac242)
Supplement: fcac242_Supplementary_Data [file fcac242_supplementary_data.zip › Supplementary_figures.pdf]

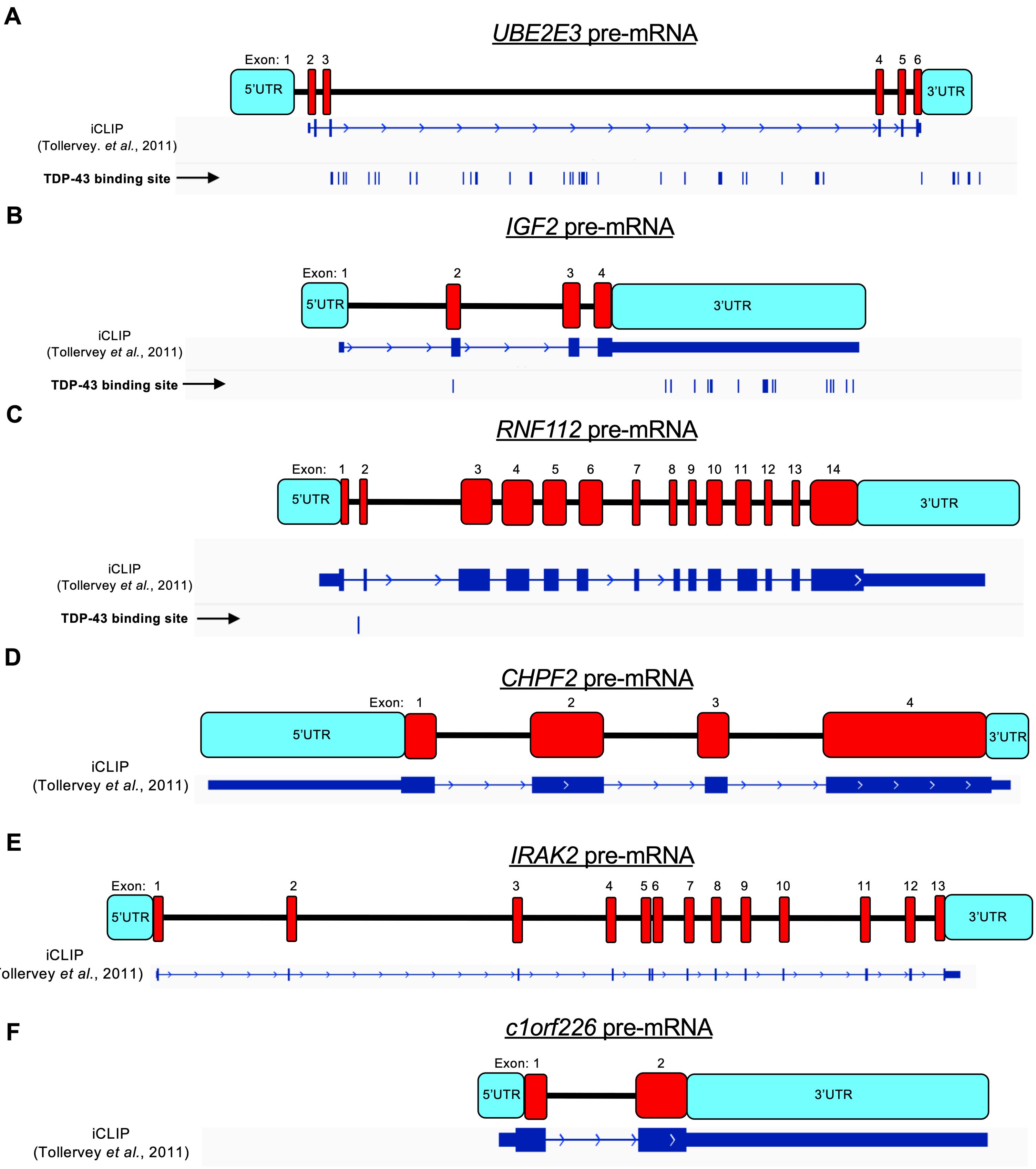

Supplementary Fig. 1 Schematic representation of six commonly regulated transcripts among TDP-43 and hnRNPs. Schematic representation of *UBE2E3* **(A)**, *IGF2* **(B)**, *RNF112* **(C)**, *CHPF2* **(D)**, *IRAK2* **(E)** and *c1orf226* **(F)** pre-mRNA (referred to the «canonical isoform» of Uniprot: Q969T4-1, P01344-1, Q9ULX5-1, Q9P2E5-1, O43187-1 and A1L170-1, respectively). Exons (red boxes) and regulatory regions (blue boxes) are reported. It is also reported the IGV genome browser's expanded view of the iCLIP analysis performed by Tollervey and collaborators (Tollervey *et al.*, 2011). For each transcript, iCLIP reads are represented as blue rods along the different gene regions. For *CHPF2*, *IRAK2* and *c1orf226* no reads were identified.

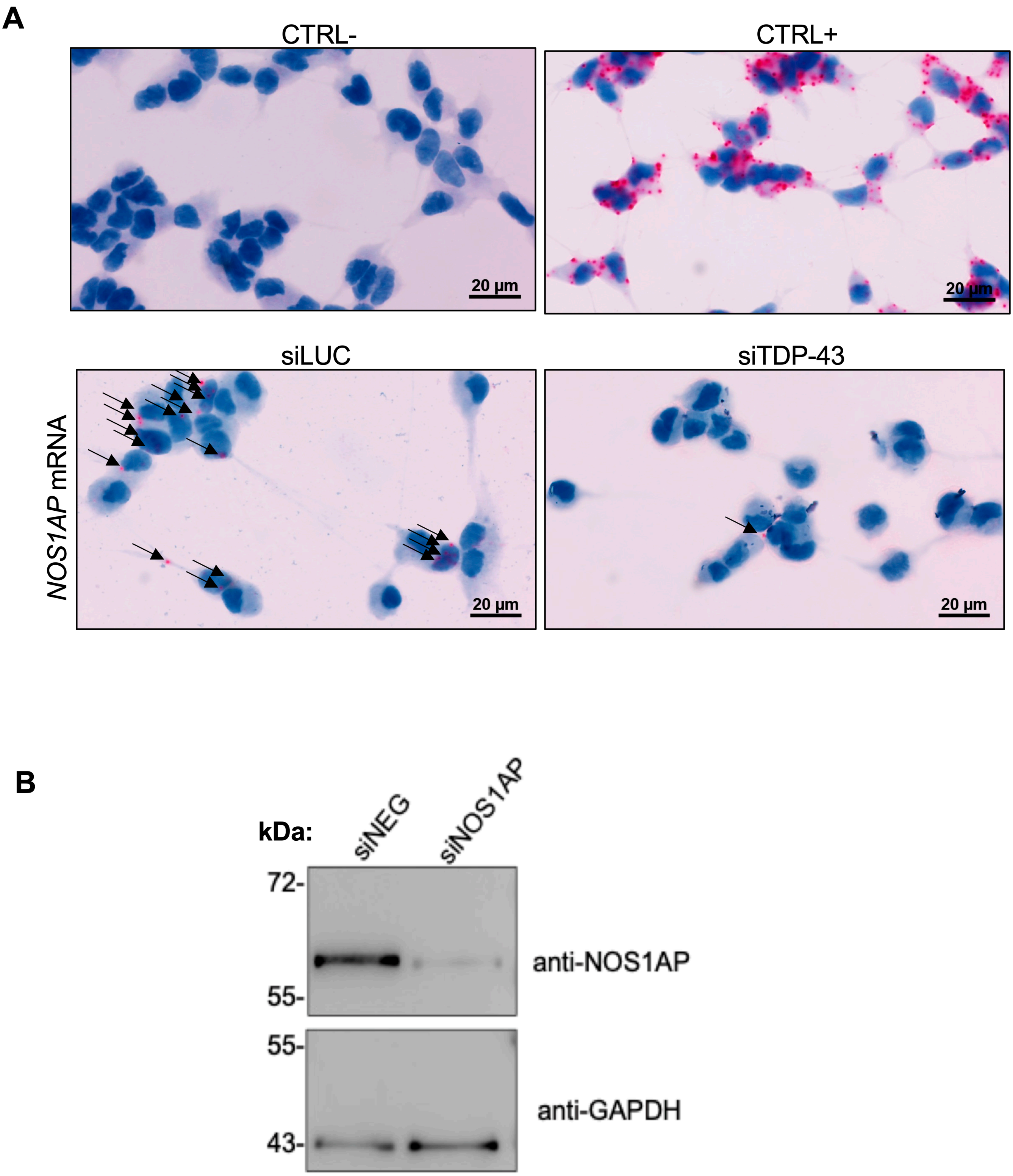

Supplementary Fig. 2 Effect of siTDP-43 and siNOS1AP treatment on SH-SY5Y cells. (A) RNAscope analysis performed on SH-SY5Y cells using *B. subtilis DapB* negative control probe (CTRL-) and human *POLR2A* positive control probe (CTRL+, red punctate dots). Detection of *NOS1AP* mRNA using RNAscope detection system in cells depleted for TDP-43 and fire-fly luciferase (control). Black arrows are used to highlight red punctate dots showing *NOS1AP* mRNA. Nuclei were stained with Gill's hematoxylin. Images were acquired with Zeiss Axioscope 5 (63X objective, original magnification). Scale bar 20 mm. **(B)** Western blotting analysis of SH-SY5Y cells treated with a pool of four non-targeting control siRNAs (siNEG) and four siRNAs against NOS1AP (siNOS1AP). Protein expression of NOS1AP was detected. GAPDH was used as loading controls.

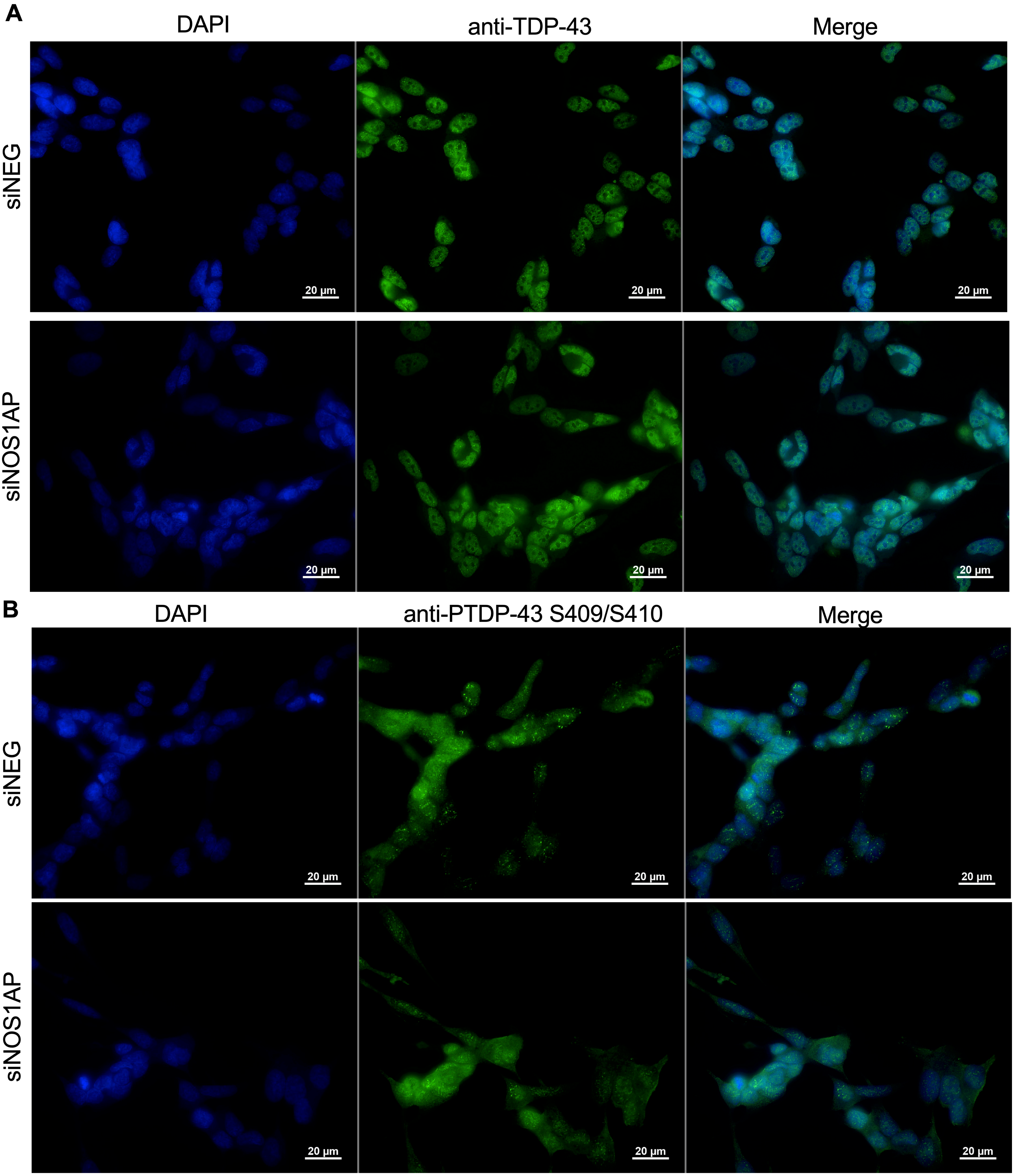

Supplementary Fig. 3 Analysis of TDP-43 localization after NOS1AP depletion. Immunofluorescence analysis of **(A)** TDP-43 and **(B)** P(phosho) TDP-43 S409/S4010 expression in SH-SY5Y cells treated with a pool of four non-targeting control siRNAs (siNEG) and four siRNAs against NOS1AP (siNOS1AP). Target proteins were labeled with Alexa-488 conjugated antibody (green signal) and nuclei were counterstained with DAPI (blue signal). Merged channels were also provided. Images were acquired with Zeiss Axioscope 5 (63X objective, original magnification). Scale bar 20 mm.

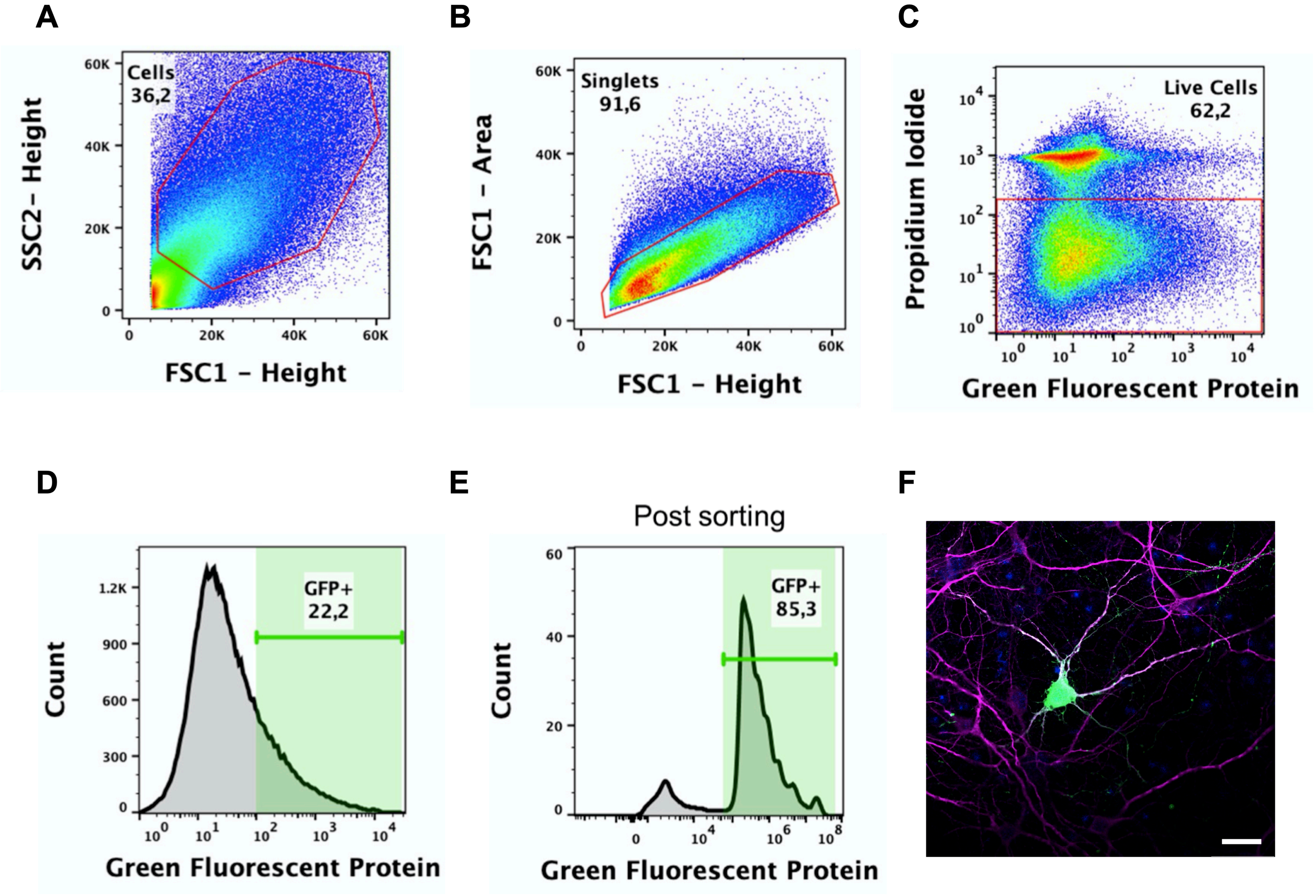

Supplementary Fig. 4 Gating strategy example for cortical primary culture cell purification. Cells are sorted by high-speed cell sorting (Moflo Astrios EQ) and checked on the Cytoflex Instrument. Transfected cortical cultures exited with 488 nm blue laser, were sorted on the basis of their **(A)** physical parameters (forward-scattered light, FSC, and side-scattered light, SSC); **(B)** singlets; **(C)** propidium iodide negative (live cells) and **(D)** Green Fluorescent Protein (GFP) intensity. (**E**) Purity check; **(F)** GFP-transfected neuron probed with the neuronal marker MAP2 (magenta). Scale bar 20 μM

A

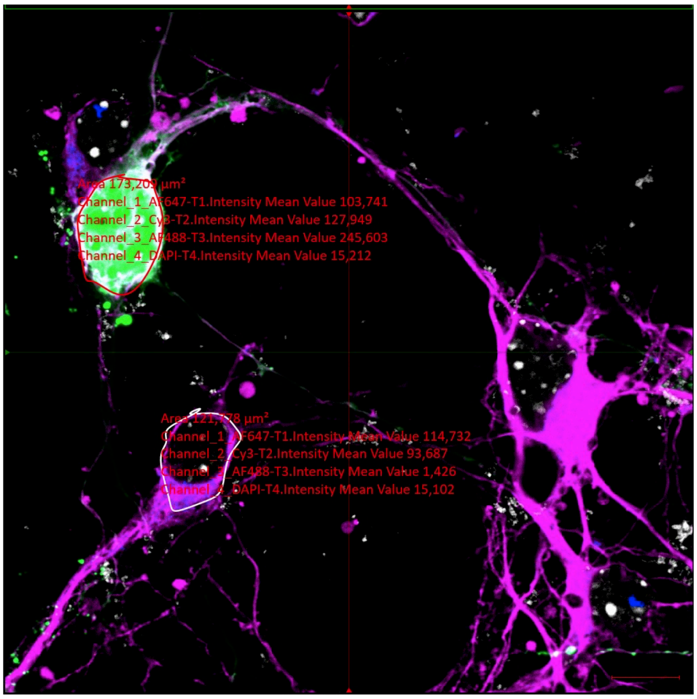

B

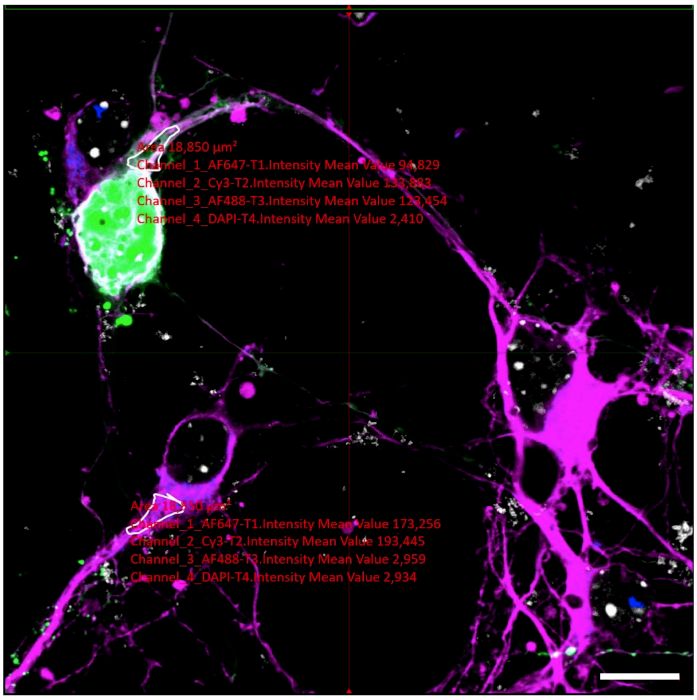

Supplementary Fig. 5 Detection of ROI area in neuronal cortical cultures. The identification of the **(A)** neuronal somata ROI area and **(B)** perisomata ROI area was performed using the Zeiss proprietary software Zen 2.6 Blue edition to quantify the A647 intensity mean values relative to the protein expression. Beta-tubulin was used as neuronal marker, the intensity mean values was performed comparing beta-tubulin probed cells (magenta) with (transfected) and without (not transfected, CTRL) the GFP construct (green) in the same image. Scale bar 10 μM.

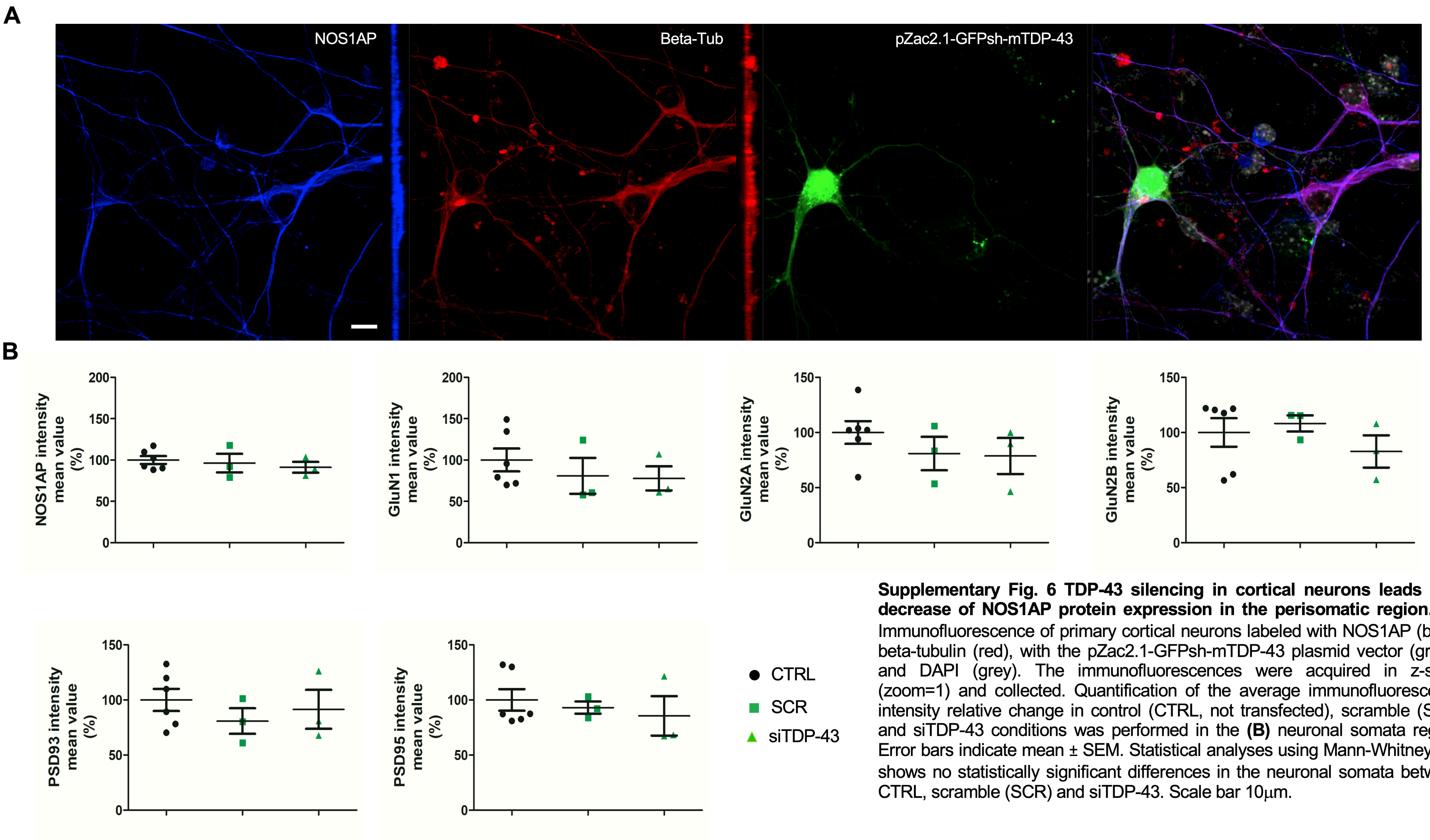

Supplementary Fig. 6 TDP-43 silencing in cortical neurons leads to a decrease of NOS1AP protein expression in the perisomatic region. (A) Immunofluorescence of primary cortical neurons labeled with NOS1AP (blue), beta-tubulin (red), with the pZac2.1-GFPsh-mTDP-43 plasmid vector (green) and DAPI (grey). The immunofluorescences were acquired in z-stack (zoom=1) and collected. Quantification of the average immunofluorescence intensity relative change in control (CTRL, not transfected), scramble (SCR) and siTDP-43 conditions was performed in the **(B)** neuronal somata region. Error bars indicate mean ± SEM. Statistical analyses using Mann-Whitney test shows no statistically significant differences in the neuronal somata between CTRL, scramble (SCR) and siTDP-43. Scale bar 10μm.

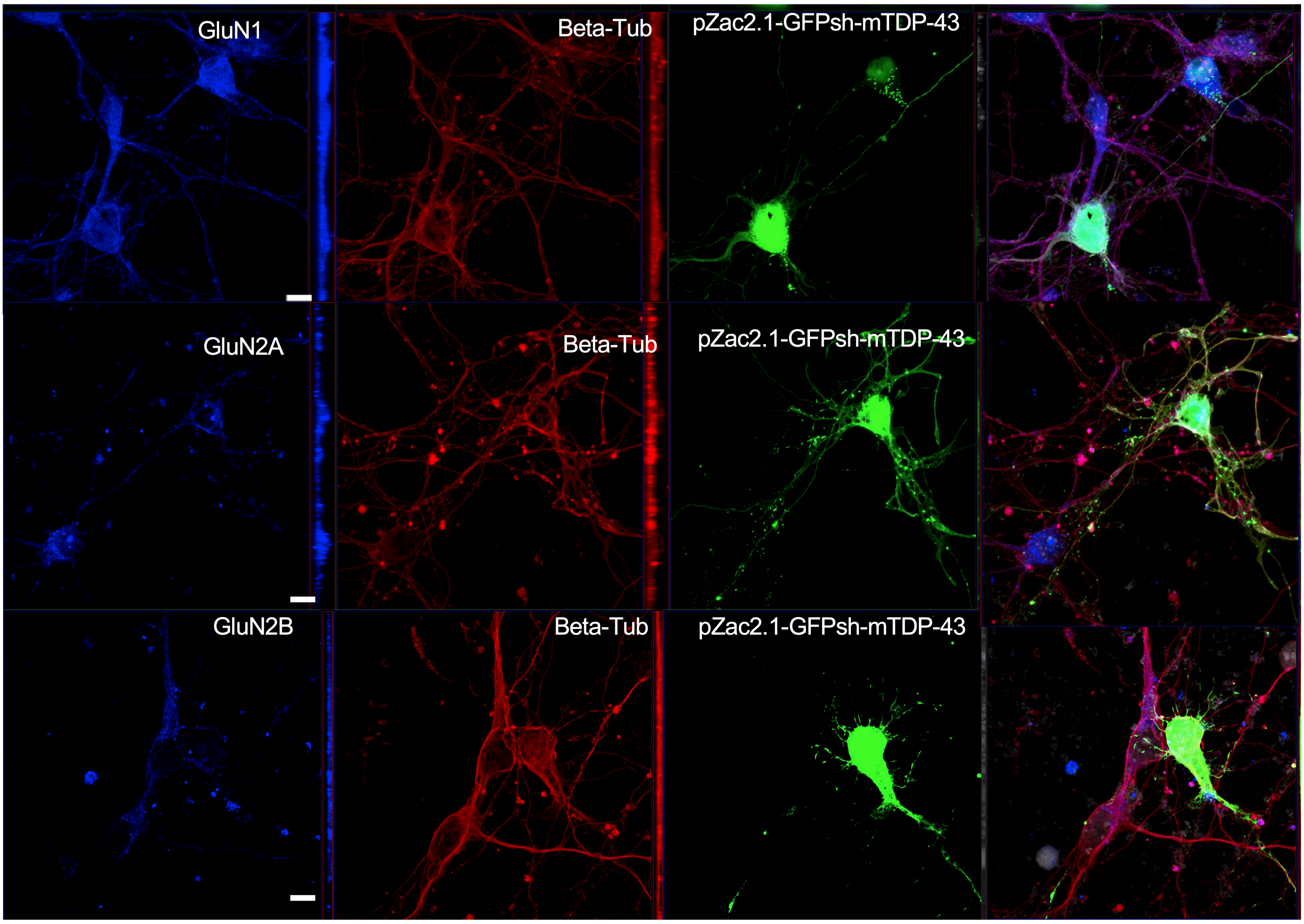

Supplementary Fig. 7 Representative immunofluorescence of primary cortical cultures. Neuronal cultures were labeled with GluN1, GluN2A, Glun2B (blue), beta-tubulin (red), the pZac2.1-GFPsh-mTDP-43 construct (green), and DAPI (grey in merge). Beta-tubulin was used to identify neurons. The immunofluorescences were acquired in z-stack (zoom=1) and collected. Scale bar 10 µM.

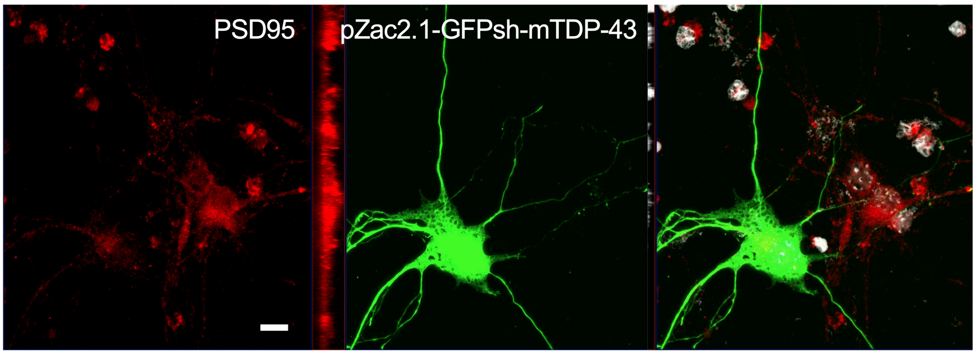

Supplementary Fig. 8 Representative immunofluorescence of primary cortical cultures. Primary cortical cultures were labeled with PSD95 (red), the pZac2.1-GFPsh-mTDP-43 construct (green), and DAPI (grey in merge). The immunofluorescences were acquired in z-stack The immunofluorescences were acquired in z-stack (zoom=1) and collected. Scale bar 10 µM.

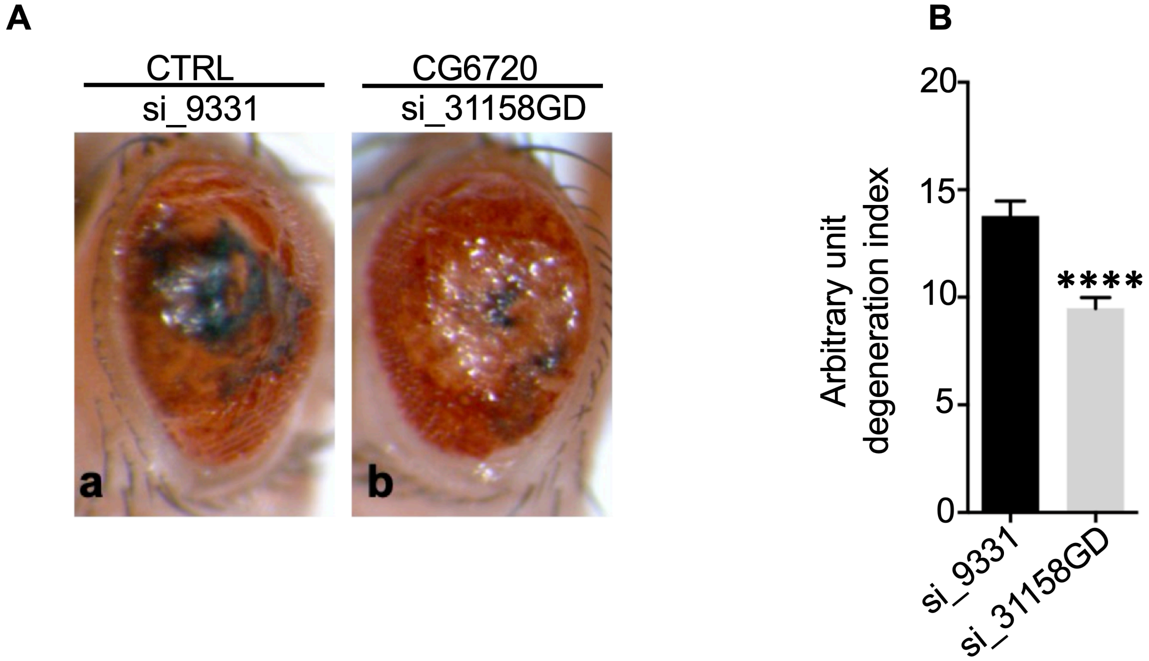

Supplementary Fig. 9 Effect of downregulating the fly homologue of UBE2E3 on TDP-43 (TBPH) toxicity in the fly eye. (A) Eye phenotype of flies expressing UAS TBPH and siRNA for CG6720. Expression of TBPH induced degeneration in Drosophila eye and the degenerative phenotype were rescued by the co-expression of siRNA silencing gene CG6720: (a) control with unrelated siRNA against GFP: GMR-Gal4,UAS-TBPH/ si_9331, (b) siRNA for CG6720 VDRC GD library: GMR-Gal4,UAS-TBPH/ si_31158GD. **(B)** Eye phenotype quantification: arbitrary units of eye degeneration index. The co-expression of siRNA silencing CG6720 rescued TBPH induced eye degeneration. Statistical analysis was performed with Prism (GraphPad) version 6.0: si_9331 (n = 52), si_31158GD (n = 48). Mann-Whitney was applied as statistical test. Values were displayed as mean ± SEM. Statistical significance displayed as: ****$P < 0.0001$.
